# Royal Decree: Gene Expression in Trans-Generationally Immune Primed Bumblebee Workers Mimics a Primary Immune Response

**DOI:** 10.1371/journal.pone.0159635

**Published:** 2016-07-21

**Authors:** Seth M. Barribeau, Paul Schmid-Hempel, Ben M. Sadd

**Affiliations:** 1 Experimental Ecology, Institute of Integrative Biology, ETH Zürich, Zürich, Switzerland; 2 Department of Biology, East Carolina University, Greenville, North Carolina, United States of America; 3 School of Biological Sciences, Illinois State University, Normal, Illinois, United States of America; Chang Gung University, TAIWAN

## Abstract

Invertebrates lack the cellular and physiological machinery of the adaptive immune system, but show specificity in their immune response and immune priming. Functionally, immune priming is comparable to immune memory in vertebrates. Individuals that have survived exposure to a given parasite are better protected against subsequent exposures. Protection may be cross-reactive, but demonstrations of persistent and specific protection in invertebrates are increasing. This immune priming can cross generations ("trans-generational" immune priming), preparing offspring for the prevailing parasite environment. While these phenomena gain increasing support, the mechanistic foundations underlying such immune priming, both within and across generations, remain largely unknown. Using a transcriptomic approach, we show that exposing bumblebee queens with an injection of heat-killed bacteria, known to induce trans-generational immune priming, alters daughter (worker) gene expression. Daughters, even when unexposed themselves, constitutively express a core set of the genes induced upon direct bacterial exposure, including high expression of antimicrobial peptides, a beta-glucan receptor protein implicated in bacterial recognition and the induction of the *toll* signaling pathway, and *slit-3* which is important in honeybee immunity. Maternal exposure results in a distinct upregulation of their daughters’ immune system, with a signature overlapping with the induced individual response to a direct exposure. This will mediate mother-offspring protection, but also associated costs related to reconfiguration of constitutive immune expression. Moreover, identification of conserved immune pathways in memory-like responses has important implications for our understanding of the innate immune system, including the innate components in vertebrates, which share many of these pathways.

## Introduction

Parasites, broadly construed to include both macro- and micro-parasites, are ubiquitous and can cause significant damage to their hosts. As a consequence, parasites represent a major selective force for any organism [1]. Hosts, in turn, have adaptations that prevent parasite establishment and reduce the costs of having an infection [2–4]. These adaptations, which can be broadly viewed as elements of a defense system, notably including the immune response, range in their specificity, their mode of action, and the nature of regulation [2, 5]. As investment into immunity is costly on multiple levels [6], the most efficient investment into immunity will be a function of the prevailing pressure from parasites (likelihood of encounter and virulence) and demands imposed by other life-history traits. On an ecological scale, there will therefore be a benefit to a plastic adjustment of immune investment based on the "perceived" risk of parasitism, if this risk can be judged to sufficient accuracy. The perception may be related to ecological conditions, such as crowding [7], but may also result from prior immunological experience with parasites. In particular, hosts can encounter the same parasites multiple times within their lifetime, and across generations, with prior encounters being predictive of future risk. If hosts encounter the same parasite repeatedly, some form of memory will be adaptive, as it would improve resistance to that same parasite upon re-exposure and thus decrease detrimental effects of infection, will be adaptive.

The best-studied and classic example of an adjustment in immune responses in relation to a prior parasite exposure is the adaptive immune system of vertebrates. The adaptive immune system, which produces specific and long-lasting protection against subsequent exposure to the same parasite, is based on a repertoire of specialized lymphocytes and its molecular underpinnings are well characterized [8]. There is growing evidence that functionally comparable processes may exist in other organisms including invertebrates [9], plants [10, 11] and even bacteria [12]. To avoid mechanism-based confusion in terms, this phenomenon in invertebrates is referred to as 'immune priming'. Astonishingly, induced protection against parasites in these systems can traverse generations, a phenomenon known as trans-generational immune priming [10, 13, 14].

The molecular understanding of immune priming outside of the adaptive immune system of vertebrates is still in its infancy. Some progress has been made in understanding these mechanisms in insects [15, 16], snails [17], and plants [18–20]. Invertebrates are particularly important to understand in this regard as they share a number of conserved characteristics of the innate immune system with vertebrates, including humans [21, 22]. The potential for these innate immune components to exhibit a memory-like response is an intriguing possibility [23, 24]. While invertebrates may serve as a valuable model for understanding memory-like phenomena produced solely by innate immune system, the mechanisms remain largely enigmatic. Studies have identified the role of the *toll* pathway and phagocytosis within an individual’s life[16] in *Drosophila melanogaster*; and cellular mechanisms seem important for immune priming in mosquitoes[25] and snails [17].

Here we investigate patterns of gene expression underlying the phenomenon of trans-generational immune priming in a social insect, the bumblebee *Bombus terrestris*. In social insects, such as bumblebees, temporal and spatial overlap of worker offspring and their mothers will mean that they are faced with a parasite threat that can, with a high probability, be predicted from the mother’s prior immunological experience. *Bombus terrestris*, is a model of ecological host-parasite interactions that shows a specific immune response [26–29], and within-individual [30, 31] and trans-generational [32, 33] immune priming. Daughters of bacterial-challenged queens show elevated antibacterial responses, but pay costs in terms of increased susceptibility to distinct parasites [30, 34]. The mechanisms underlying these phenotypes are unknown.

**Fig 1:**
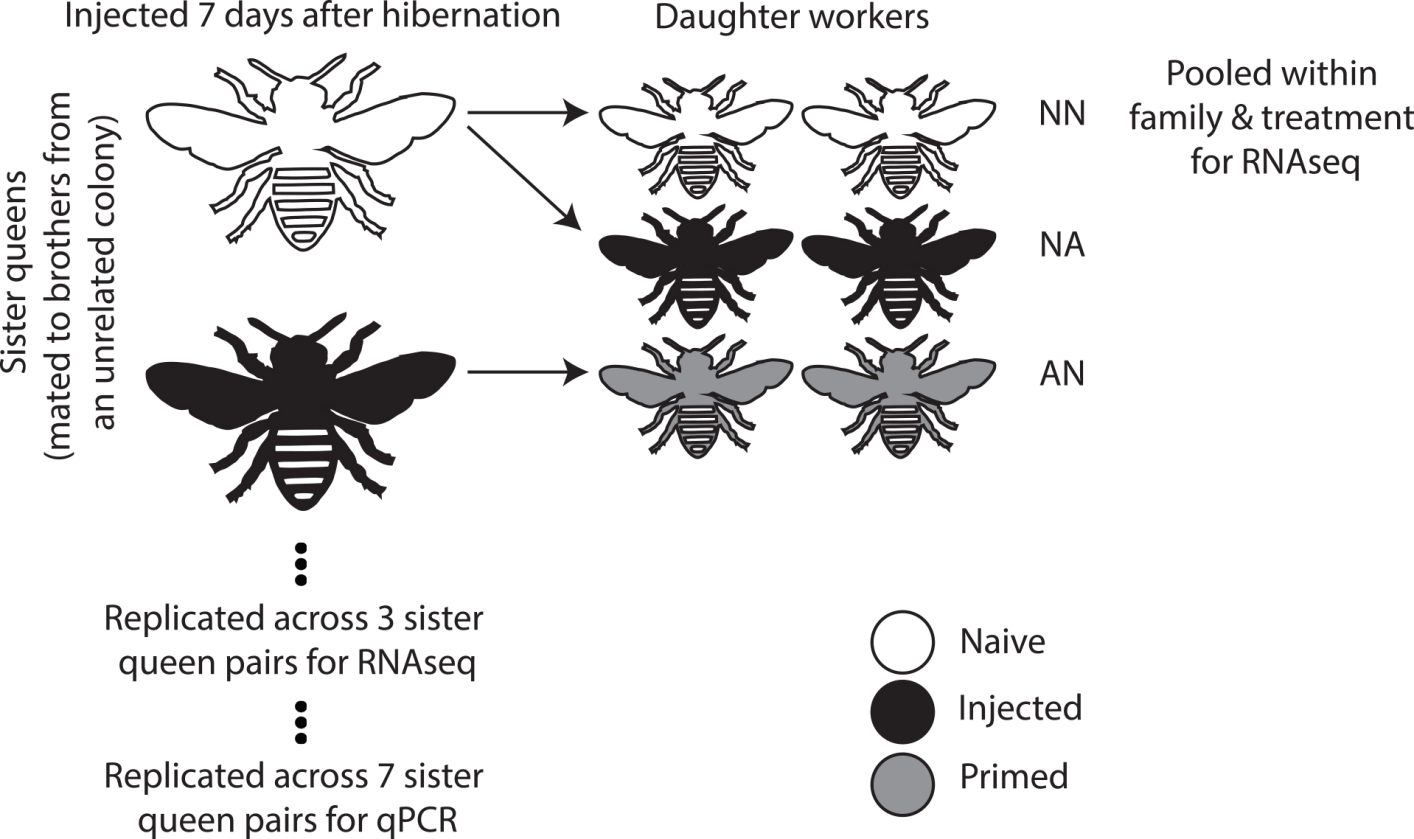
Diagram of our experimental design. We mated full sister queens to brothers from an unrelated colony to create colony blocks with limited genetic differences between them. These sisters were then injected with heat-inactivated bacteria or left unchallenged as controls. The resulting daughters were then either left unchallenged or given an injection as above and the RNA pooled from two individuals per colony by treatment group for RNAseq. We used additional colonies to confirm our findings with qPCR.

We injected *B*. *terrestris* queens with a heat-inactivated inoculum of the Gram-positive bacterium *Arthobacter globiformis*, to replicate conditions of previous studies demonstrating trans-generational immunity in this system [33, 34], To gain some insight into the molecular foundations of observed trans-generational immunity, we measured genome-wide expression of subsequently produced naïve daughters (*Arthrobacter*-Naïve [AN] treatment) relative to the expression of naïve daughters born from unchallenged mothers (Naïve-Naïve [NN] treatment). We further contrast this memory response comparison with the immune response of daughters that are exposed to the bacterial challenge, but whose mothers were naïve (Naïve-*Arthrobacter* [NA] treatment). These comparisons took place within and between matched colony blocks to control for differences in genetic background (a matched block being colonies of sister queens that each had been mated to brothers from a different unrelated colony) (Fig 1). An unmatched comparison of offspring from naïve mothers (NN) and those from a mother receiving a procedural control injection of sterile saline solution was used to establish if wounding *per se* could be responsible for any trans-generational alterations of immune gene expression.

## Results

We found that no genes were significantly differently expressed between naïve daughters of naïve mothers (NN) and naïve daughters of procedural control mothers injected with sterile saline. We thus feel confident that exploring the expression profile of trans-generationally primed workers (AN) relative to naïve workers from naïve mothers (NN) gives us an insight into expression changes resulting from maternal exposure to a bacterial-based inoculum. Subsequent RNASeq and confirmatory qPCR investigating trans-generational immune priming were carried out in matched colony blocks of sister queens each mated to brothers from an unrelated colony. However, we did not have enough successful colonies from sterile saline injected queens to use their workers in this matched RNAseq design. Therefore, we analyzed naïve offspring from these queens, irrespective of colony block, for the expression of 21 immune genes by qPCR (S1 Table). We found that even when using fairly liberal t-tests without controlling for multiple testing, no genes were significantly differently expressed between naïve daughters of naïve mothers (NN) and naïve daughters of mothers injected with sterile saline.

**Fig 2:**
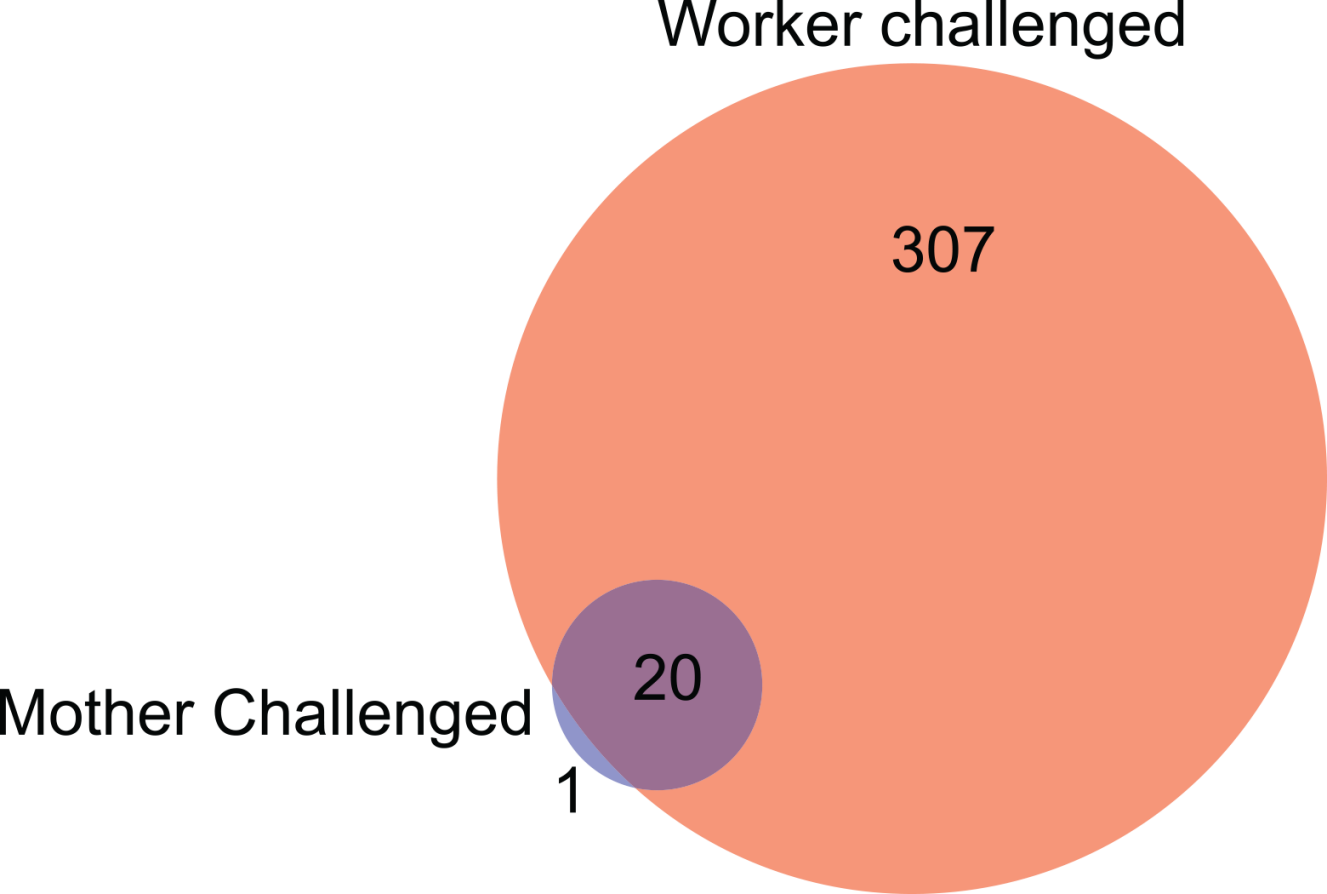
The number of differentially expressed genes in naïve worker offspring of mother queens that were injected with heat killed Gram-positive bacterium (*Arthrobacter globiformis*) (trans-generational immunity treatment; AN), and worker offspring from naïve mother queens but themselves exposed to an immune stimulus of *A*. *globiformis* (induced immune response condition; NA). The expression of these genes is measured relative to that of naïve worker offspring of naïve mothers (NN).

Whole genome expression, as measured by mRNA sequencing on the Illumina HiSeq platform revealed that when workers from un-primed, naïve, mothers were directly exposed to the bacterial inoculum (NA) they responded with significant differential expression of 327 genes (S2 Table). Naïve workers from primed mothers (AN) significantly altered the expression of only 21 genes (S3 Table), but 20 of these are shared with the direct induced response (NA) (Fig 2). These shared genes (Fig 2) include all known bumblebee antimicrobial peptides (*abaecin*, two *apidaecins*, *defensin*, *hymenoptaecin*) and a number of additional known immune genes such as *battenin*, *laccase-2*, *slit-3*, and *yellow* [35–38]. Two genes were differentially expressed in both experimental groups but in opposite directions. The venom protease LOC100651916 and an unknown bee specific gene LOC100643115 were more highly expressed in the AN and NA workers, respectively. The only gene differentially expressed in the primed condition, but not under direct induction, codes for LOC100644816, a 53aa hydrophobic (58.49% of residues) peptide with homology to Mast Cell Degranulating Peptide (MCDP) from another bumblebee, *B*. *pennsylvanicus*.

**Fig 3:**
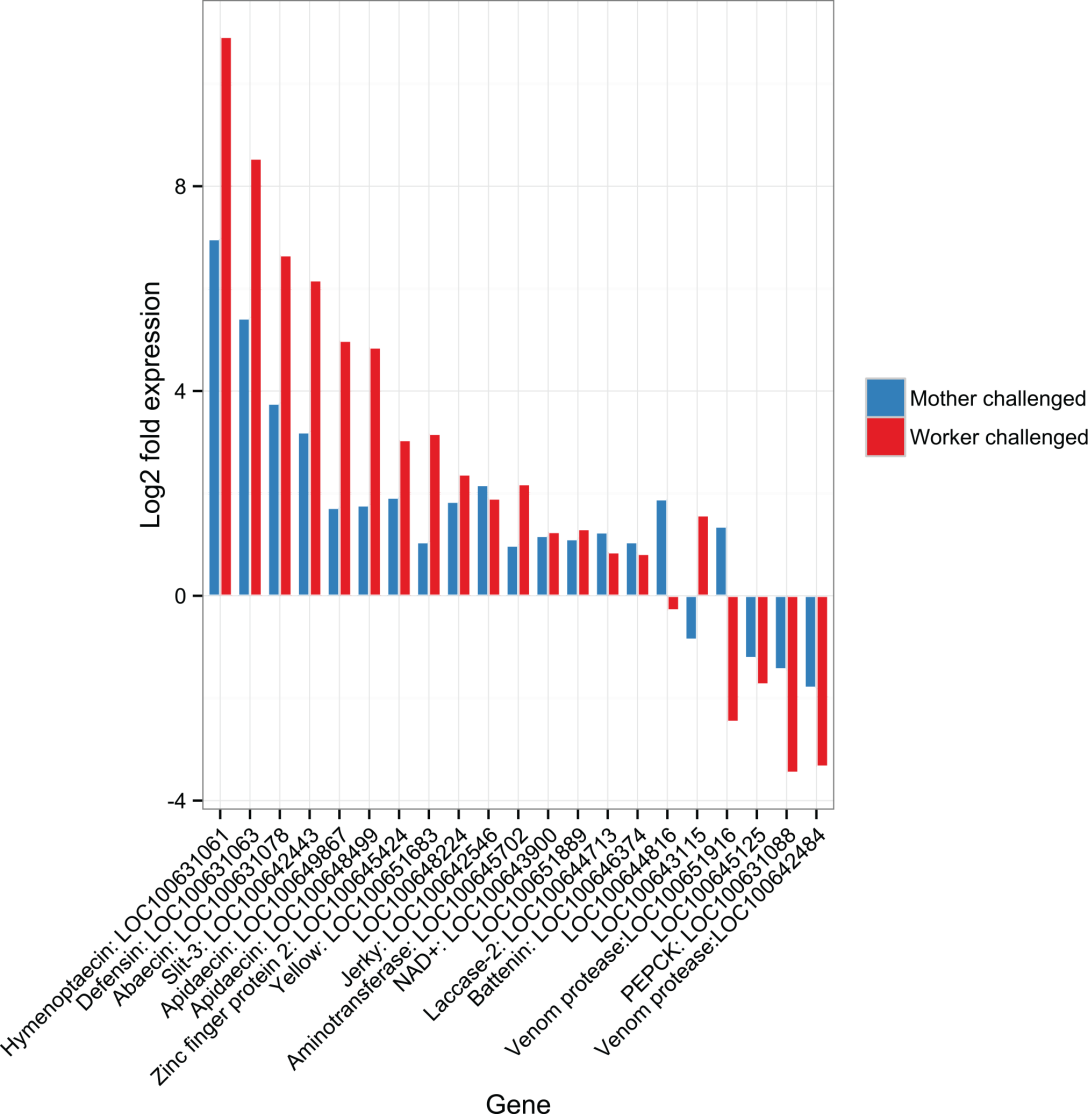
Log 2 fold expression based on RNAseq data (relative to naïve worker offspring from naïve mother queens) for all genes that are significantly differentially expressed in the trans-generational priming condition (naïve offspring of bacteria exposed mothers, blue). We also show the expression of directly bacterially exposed workers from naïve mothers (red) to demonstrate the similarity of the induced response to a direct challenge to the signature of trans-generational immunity. All differentially expressed genes here are also significantly differentially expressed upon direct exposure, except for LOC100644816, which encodes for mast cell degranulating peptide. qPCR confirmation of these results can be found in S1 Fig.

We confirmed the patterns determined by the whole genome transcriptome approach (Fig 3) by targeted qPCR of a suite of immune genes (S4 Table). Our qPCR results agree with our transcriptomic results for all tested genes. This included the high constitutive expression of the antimicrobial peptides and additionally of a beta-glucan receptor protein (BGRP, S1 Fig, S5 Table) in naïve offspring of immune-primed mothers (AN). There was a trend for higher BGRP expression in the transcriptome of workers from primed mothers, but this was not significant after correction for multiple testing (*P* < 0.01 before correction, *P* = 0.072 for false discovery rate adjusted p-value). Differential expression of this receptor may be particularly relevant as it can trigger the *toll* signaling pathway and downstream antimicrobial peptide production[39].

We identified a number of different isoforms for putative immune response genes, including for antimicrobial peptides (S2, S3, S4, S5 and S6 Figs: *abaecin* [LOC100631078], 4 isoforms; both *apidaecins* [LOC100649867], 2 isoforms, and [LOC100648499], 3 isoforms, aminopeptidase [LOC100645702], tetraspannin [LOC100651747], a venom protease [LOC100651916], an uncharacterized protein shared only within honeybees and bumblebees [LOC100645125], and a novel gene [NC_015763.1:3848320–3855802] with sequence homology to *A*. *mellifera* cuticular protein 14. We also identified two *dscam* like genes with multiple isoforms (S2, S3, S4, S5 and S6 Figs; LOC100644003, 12 isoforms; LOC100649765, 9 isoforms). Among the significantly differentially expressed genes, isoform transcript abundance did not vary significantly among conditions.

## Discussion

We find that offspring workers that had never been exposed themselves ("naive workers"), but whose mothers were exposed to bacterial immune elicitors, express a strikingly exposure-like immune response, as compared to offspring workers from naïve mothers. In fact, all but one of the differentially expressed genes in this priming condition are shared with workers that were directly immune stimulated with the same bacterium. This indicates a major reconfiguration of the constitutively expressed immune gene profile, and is one that could confer benefits in the face of repeated parasite exposures, but may also result in the costs previously described when there is mismatch between the maternal parasite environment and the offspring parasite environment [34]. These results give us an insight into the innate immune-related molecular pathways involved in invertebrate immune priming across generations.

Among the differentially expressed genes, all of the antimicrobial peptides are upregulated. It is particularly noteworthy that these are the final product of the immune response, indicating an immediate readiness of defense in trans-generationally primed individuals. We also find increased expression in a number of other immunologically important genes including *yellow* and *laccase-2*, which are involved in the melanization response [35, 36], *battenin*, the *D*. *melanogaster* homolog of which (*CLN3*) regulates *JNK* signaling [38], and *slit-3* which is induced upon bacterial challenge in honeybees and the leaf-cutting ant *Atta cephalotes* (a.k.a. *IRP30*)[37]. We also found that a beta-glucan receptor protein (BGRP) was more highly expressed in naïve workers of primed queens. BGRPs induce the *toll* signaling pathway in invertebrates [39]. The only gene that was differentially expressed in the primed condition, but not in workers directly exposed to bacterial elicitors, was a gene with homology to mast cell degranulating peptide (MCDP), which is has been found in venom [40]. MCDP, neuro- and immunotoxic, is named for its degranulating effect on vertebrate granulocytes [40, 41]. Whether this peptide also affects invertebrate granulocytes (a class of haemocytes) that are important for phagocytosis [42] is unknown. Interestingly, intra-generationally primed *Drosophila melanogaster*, utilize the *toll* pathway and phagocytosis, but not antimicrobial peptides [16] that appear to play an important role here.

The down syndrome cell adhesion molecule (*dscam*) is implicated in immune defenses, and because of its ability to produce prodigious numbers of isoforms [43] has been proposed as a possible mechanism for specific immune memory [44]. We detect two *dscam* like genes that produce multiple isoforms. However, neither these genes nor their isoforms are differentially expressed in the priming condition or in workers that are directly exposed to the bacterium. While this does not rule out a role for *dscam* isoforms in immune priming, it suggests that differential expression of isoforms is not a major component of trans-generational antibacterial immune priming in this system.

On an individual level, wounding alone can lead to significant changes in gene expression [45], and it is conceivable that a general primed response could result from cuticular trauma in mothers. An effect of maternal experience of trauma alone influencing offspring immune phenotype is interesting in itself, but overall our results suggest that this is not the case. Our RNASeq and follow up qPCR based on comparisons between AN and NN offspring are not sufficient to disentangle effects of maternal exposure to a bacterial elicitor and wounding on the production of a primed offspring phenotype. However, our targeted survey of immune gene expression, including the apparently important antimicrobial peptides of bumblebees, in offspring from naïve mothers and mothers receiving a procedural control injection of sterile saline provide evidence that maternal wounding alone is not responsible for changes in offspring immune phenotype. Rather, it appears that it is exposure to a bacterial elicitor that triggers the trans-generational response. It is possible, however, that some of the additional genes seen differentially expressed in the direct exposure group (NA) compared to the primed group (AN) are the result of a response to wounding within individuals.

A prior study on the bumblebee system demonstrated that trans-generational priming of antibacterial activity takes place prior to egg laying, but persists through the development of offspring, even in the absence of the priming mother [33]. The elevated constitutive gene expression into adulthood, which we show here, is further testament to the persistence of the trans-generational priming in the innate immune system, despite developmental rearrangements, including the process of metamorphosis in holometabolous insects. Evidence in insects of such elevated persistent constitutive expression of immune-related genes that is precipitated by immune experiences in prior generations is important beyond a demonstration of the underlying mechanistic foundations of trans-generational immunity. It will also have important consequences for the fitness costs associated with this phenomenon, which will influence the conditions under which it may be expected to evolve and be maintained by selection. Elevated immune investment may come at a cost to an organism through resource trade-offs that can affect phenotypic traits such as developmental time, size, etc. [46]. Higher constitutive expression of immunity in naïve offspring may constrain their investment into other life-history traits, especially under conditions where resources are scarce. Such costs of trans-generational immunity have been demonstrated in other insect systems [47, 48]. The striking signature of gene expression related to trans-generational immunity is also likely to underpin other related costs, including increased susceptibility to a distinct parasite infection, as has been previously demonstrated in this system [34].

Evidence is mounting that the evolutionarily ancient innate immune system is able to retain information about immune history in both vertebrates and invertebrates [49], which translates to better defenses upon subsequent exposure. This priming effect is observed both within the lifetime of an individual and between parents and offspring. Trans-generational immune priming likely evolved as a part of parental care and investment into offspring. This may be particularly important in social insects, such as *B*. *terrestris*, where generations overlap and related individuals share an environment—including parasites—in a closed, populous, highly interactive colony. While our study does not attempt to identify the mechanisms involved in transfer of immune compounds to the offspring, a recent paper in honeybees identified the yolk protein vitellogenin as playing a role in binding and transferring bacterial cell components to eggs [50]. Here we find that trans-generationally primed workers, even if not infected themselves, increase transcription of antimicrobial peptides (that in part are under the control of the *toll* signaling pathway) and a key recognition protein that induces *toll* signaling. This transcriptional signature resembles an abridged version of the normal response to infection, suggesting that *B*. *terrestris* achieves trans-generational protection by sequestering the existing induced responses into prophylactic constitutive expression to prevent parasite establishment. A recent study in moths found elevated ovary expression of some immune genes in daughters of challenged mothers, hinting that these responses may even be transmitted across multiple generations [51].

Some innate immune pathways are highly conserved and are even shared between invertebrates and vertebrates. Functionality of the vertebrate innate immune system has also recently been shown to be dependent on prior immunological experience, a phenomenon referred to as trained immunity [49, 52]. An intriguing possibility is that both invertebrates and vertebrates may share similar mechanisms of underlying these innate immune memory-like responses.

## Materials and Methods

We collected queens of the bumblebee *B*. *terrestris* as they emerged from hibernation in spring 2013 in northern Switzerland and maintained them under standard colony establishment conditions [26]. Collections of this species, which is not protected, took place on private land with the permission of the owners. All of the colonies used for this experiment were microscopically checked for common infections twice and found to be clear of identifiable infection by the trypanosome *Crithidia bombi*, microsporidian *Nosema bombi*, or the Neogregarine *Apicystis bombi*. On their production by the colonies, young queens (gynes) were removed and mated to males from unrelated colonies. We deliberately used second generation colonies to exclude unknown maternal effects outside of our treatments, which could have been present in field caught queens, and in addition to control genetic background. We designed the matings such that sister queens from one colony were mated to males all derived from a single colony to produce comparable genetic backgrounds for matching across treatments (sister queens each mated to brothers from the same unrelated colony). Five days after mating, we hibernated the queens for 48 days at 4°C. Seven-days after removal from hibernation, exposed queens were injected with 2 μl of 10^8^ colony-forming-units/mL of *Arthrobacter globiformis* (DSM 20124) that had been heat inactivated by heating at 95°C for 5 min, washed three times and resuspended in ringer saline solution. Procedural control queens were injected only with 2 μl of sterile ringer saline solution (these queens did not produce enough colonies within the matched genetic backgrounds for inclusion within the RNASeq design, but were used in separate comparisons, see below). Naive, unexposed queens were handled similarly but not injected. We then allowed queens to found colonies in the lab. Emerging adult worker offspring from naïve queens were uniformly distributed to a naive group (NN) or an induced treatment (NA). In the induced treatment, five-days post-eclosion daughters received an injection of 2 μl of 10^8^ colony-forming-units/mL of *A*. *globiformis* prepared as above (NA: naïve queens, *A*. *globiformis* exposed worker daughters) and were snap frozen in liquid nitrogen 24hrs after injection. Naïve group workers (NN) were handled similarly, but not injected, and frozen at the same time. Similarly, we took workers from queens that were exposed to the bacterial injection or the procedural control and handled and froze them as above (AN). We extracted RNA from the workers following the same protocols as in [26] but using whole abdomens. For RNA sequencing we used the RNA from two individual workers for each of three queens and for each treatment combination resulting in six individuals per condition (not including workers from the unmatched saline injected mothers, Fig 1). The RNA was pooled within each mother queen by treatment conditions resulting in three replicate RNAseq libraries of two pooled workers for every condition (NN, AN, NA) per matched genetic background block. To reduce ribosomal contribution the library preparation included poly-A enrichment. This also removed any bacterial contamination of the library. The library preparation and sequencing used the Illumina HiSeq 2000 platform and was carried out at the Beijing Genomics Institute.

After removing adapters and poor quality reads we mapped the reads to the *B*. *terrestris* genome [53] with Tophat2 [54] in two ways. First, using the annotated transcripts (-G option) to assess differential expression of known genes, and second, without this restriction to assess isoform variation. We identified differentially expressed genes using Cuffdiff [55, 56]. In both cases we used the current version of the *B*. *terrestris* genome (Bterr_1.0) with the accompanying gtf annotation file, although for the–G option we reduced this to coding sequence. We limited the maximum intron size to 50kb. The analyses compared the expression of naïve daughters of bacterially exposed mothers (AN) vs naïve daughters of naïve mothers (NN), and separately compared the induced response (NA) to the baseline expression of NN workers using Cuffdiff [55, 56] and cummRbund [57].

From the un-pooled offspring samples described above, to confirm RNASeq results, we also synthesized cDNA using the QuantiTect Reverse Transcription Kit (Qiagen) following the manufacturers instructions. In addition to those samples contributing to the RNASeq analysis, cDNA was synthesized from offspring of queens from three additional matched genetic background providing further AN, NA and NN samples (N = 11 for each treatment condition from a total of seven genetic backgrounds of colonies). Prior to cDNA synthesis potential DNA contamination was removed from all RNA samples using the Turbo DNA-free kit (Ambion) according to the manufacturer’s instructions. In the reverse transcribed samples, we quantified the expression of 25 immune genes relative to two invariant housekeeping genes (elongation factor 1α and ribosomal protein L13 based on their scores in geNorm, qBase plus, biogazelle) and analyzed as in [26]. Full details of these genes and their primers are in S4 Table. We used the mean difference in expression of the target gene from the composite housekeeping gene (dCt) from each colony for subsequent analyses. We transformed the mean dCt value for each gene using Yeo-Johnson transformations to improve normality and homoscedasticity and used paired t-tests within colony genetic background to assess statistical differences between NN and AN treatments and between NN and NA treatments (S5 Table).

While unmatched with regard to genetic background, the same qPCR protocol and analysis was used to compare gene expression of a subset of immune-related genes (S1 Table) in naïve offspring from naïve mothers (NN) and naïve offspring from mothers receiving a procedural control injection of sterile ringer saline.

## Supporting Information

S1 Fig Expression values (ddCt) of 25 genes of interest from the RNAseq results or *a priori* predictions of relevance to immune response relative to two invariant housekeeping genes. ~ P < 0.1, * P < 0.05, ** P < 0.01, *** P < 0.001.
(PDF)

S2 Fig Isoform structure of the antimicrobial peptide abaecin. (PDF)

S3 Fig Isoform structure of the antimicrobial peptide apidaecin 73a. (PDF)

S4 Fig Isoform structure of the antimicrobial peptide apidaecin 73b. (PDF)

S5 Fig Isoform structure of the dscam-like gene LOC100644003. (PDF)

S6 Fig Isoform structure of the dscam-like gene LOC100649765. (PDF)

S1 Table Summary statistics from analysis of qPCR data comparing naïve daughters of mothers injected with sterile saline, as a procedural control, to naïve daughters of naïve mothers (NN). (PDF)

S2 Table Differentially expressed genes between the injected daughters of naïve (NA) mothers and naïve daughters of naïve mothers (NN). (PDF)

S3 Table Differentially expressed genes between the naïve daughters of injected mothers (AN) and naïve daughters of naïve mothers (NN). (PDF)

S4 Table Genes used for confirmation qPCR of RNAseq data and their primer sequences. (PDF)

S5 Table Summary statistics from analysis of qPCR confirmation of NA vs NN and AN vs NN. Cells highlighted in yellow are significant at p < 0.05, those highlighted in blue approach significance p < 0.1.
(PDF)
