# Supplementary figures and images for: Royal Decree: Gene Expression in Trans-Generationally Immune Primed Bumblebee Workers Mimics a Primary Immune Response
Source: PLoS One. 2016 Jul 21;11(7):e0159635. doi: 10.1371/journal.pone.0159635 (PMC4956190; doi:10.1371/journal.pone.0159635)

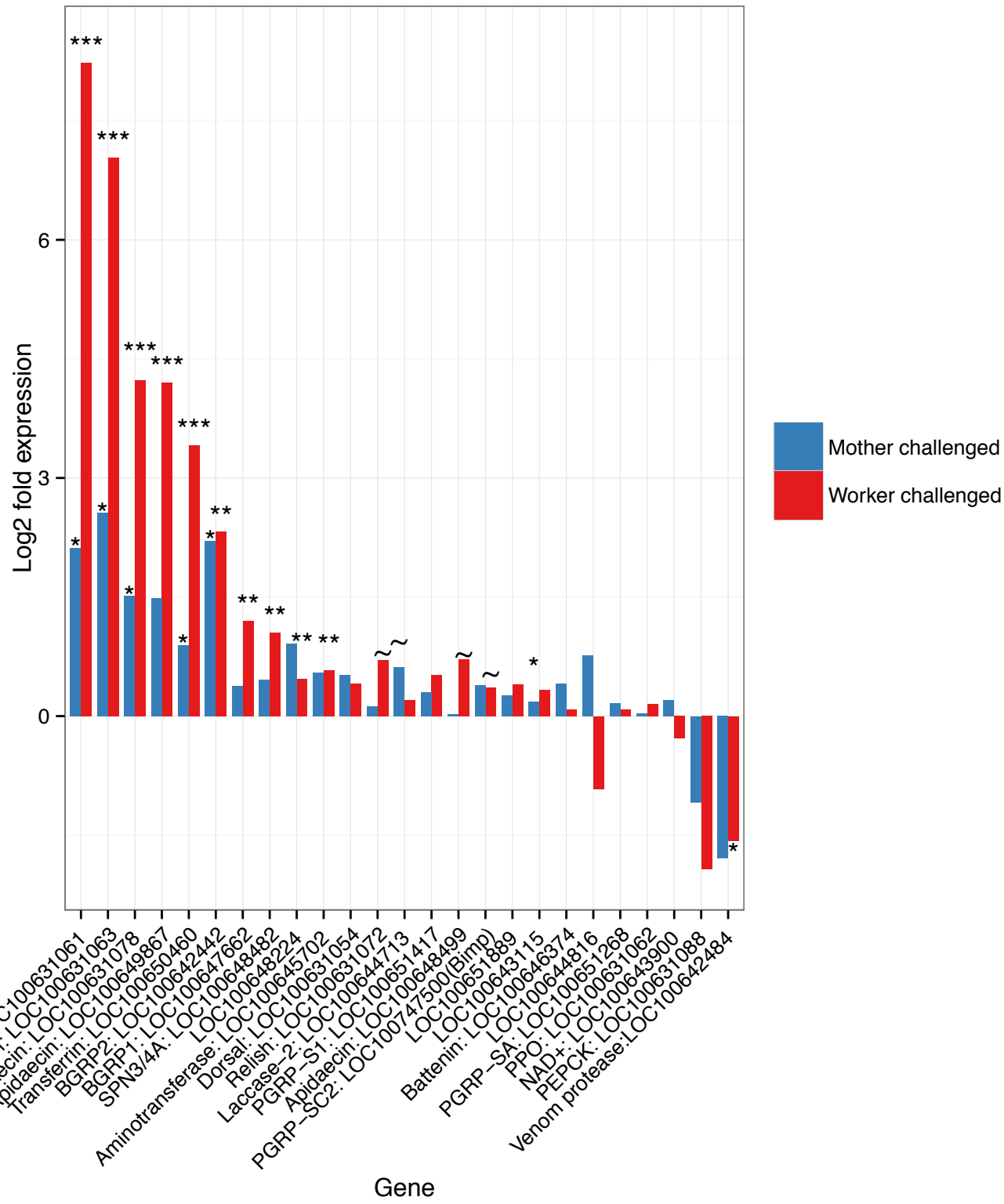

Supplement: S1 Fig — ~ P < 0.1, * P < 0.05, ** P < 0.01, *** P < 0.001. (PDF) [file pone.0159635.s001.pdf]

abaecin:  
LOC100631078

# Scaffold: NC\_015764.1

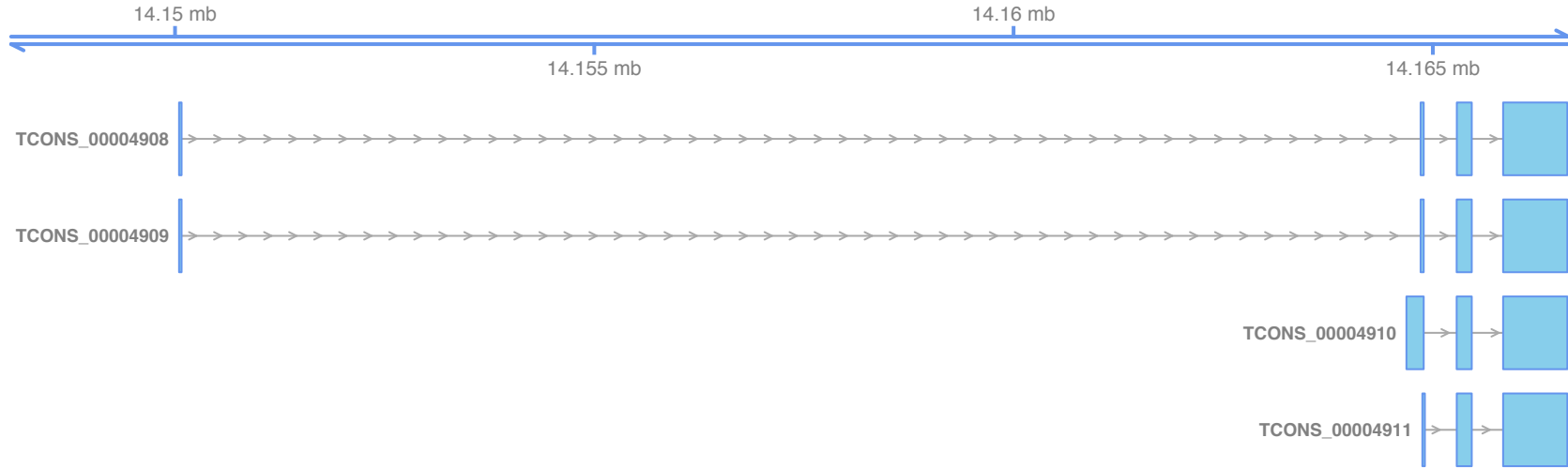

Supplement: S2 Fig — (PDF) [file pone.0159635.s002.pdf]

Scaffold: NW\_003566286.1

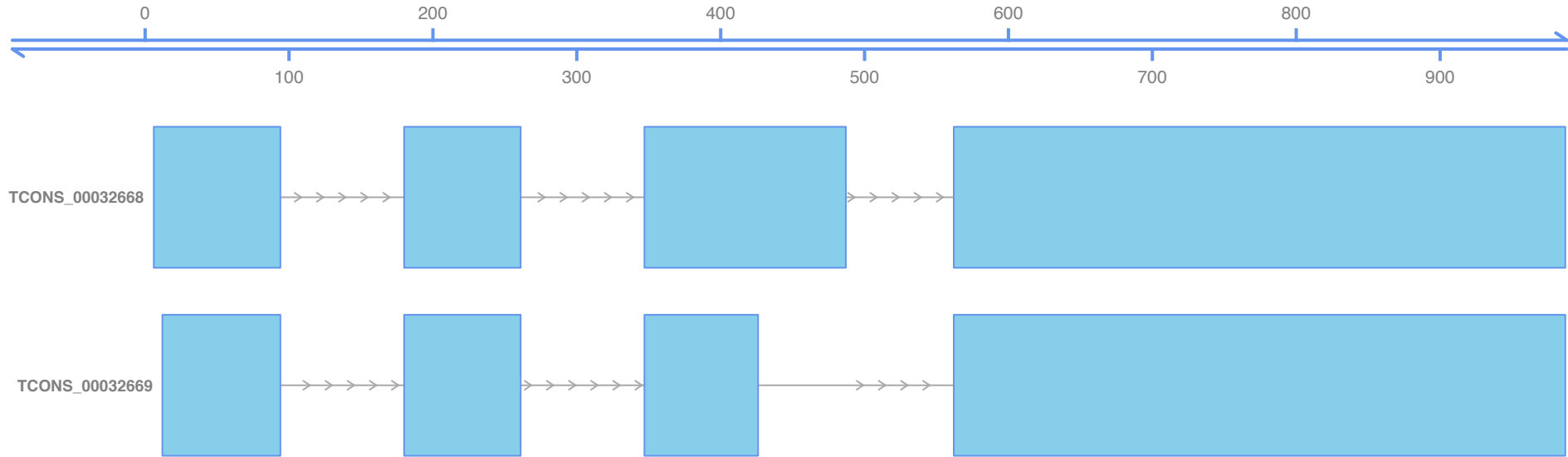

apidaecin  
73a:  
LOC100649867

Supplement: S3 Fig — (PDF) [file pone.0159635.s003.pdf]

Scaffold: NW\_003567459.1

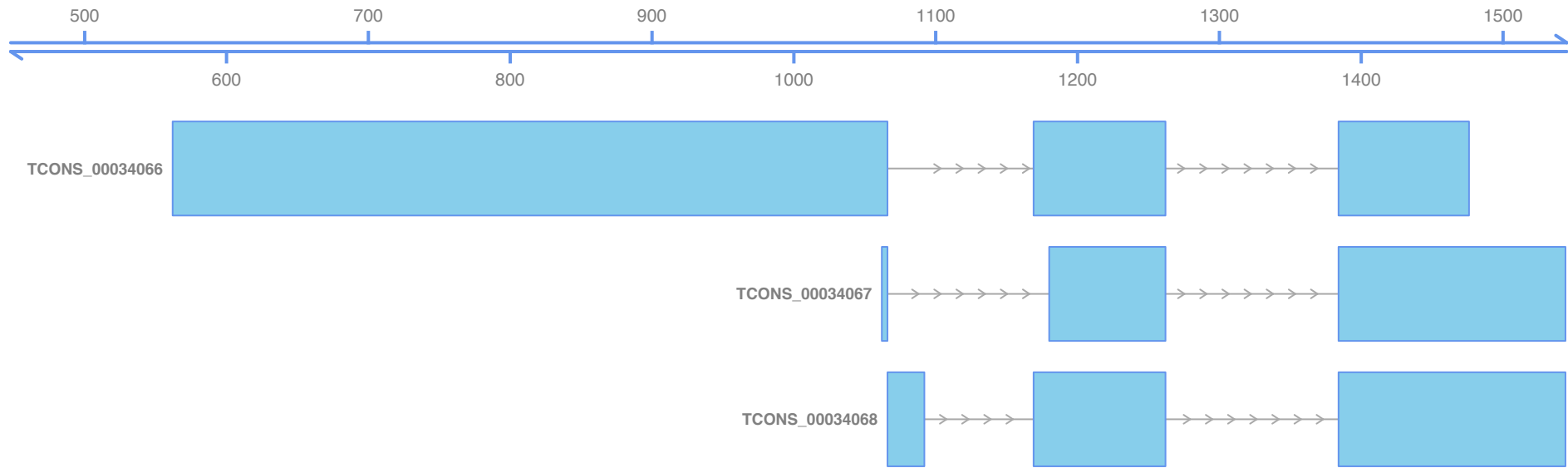

apidaecin  
73b:  
LOC100648499

Supplement: S4 Fig — (PDF) [file pone.0159635.s004.pdf]

Scaffold: NC\_015763.1

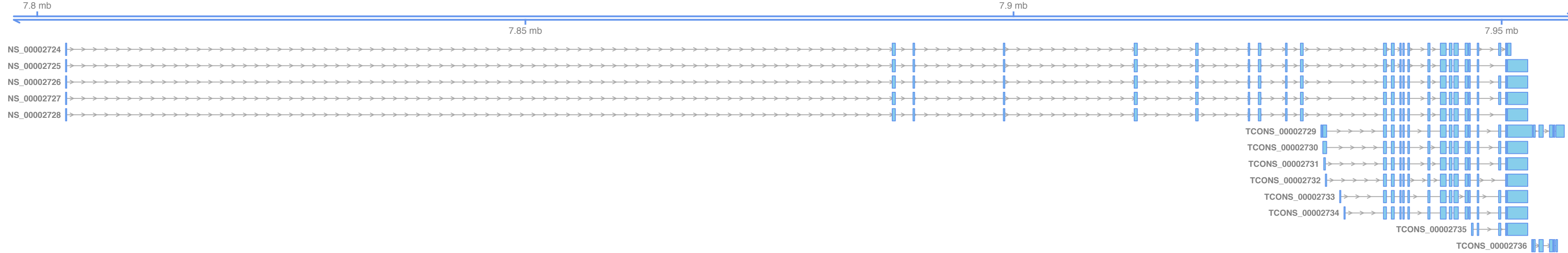

Supplement: S5 Fig — (PDF) [file pone.0159635.s005.pdf]

Scaffold: NC\_015765.1

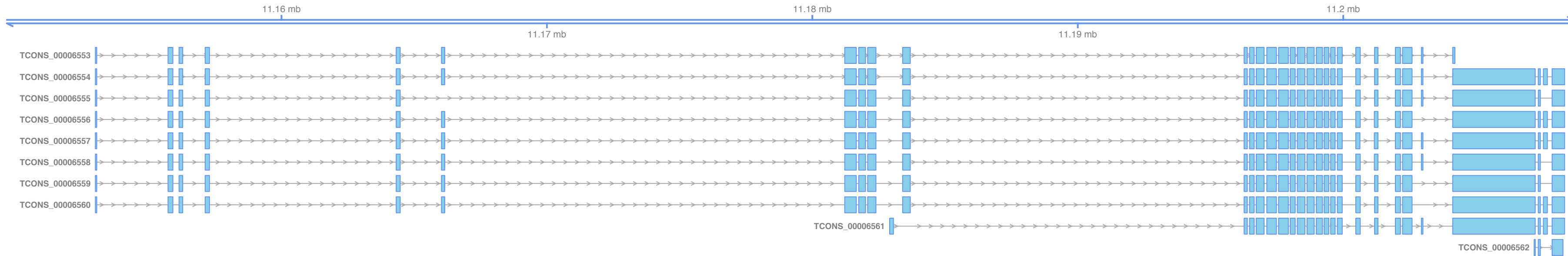

Supplement: S6 Fig — (PDF) [file pone.0159635.s006.pdf]
