# Supplementary material for: Royal Decree: Gene Expression in Trans-Generationally Immune Primed Bumblebee Workers Mimics a Primary Immune Response
Source: PLoS One. 2016 Jul 21;11(7):e0159635. doi: 10.1371/journal.pone.0159635 (PMC4956190; doi:10.1371/journal.pone.0159635)
Supplement: S1 Table — (PDF) [file pone.0159635.s007.pdf]

gene	t-statistic	p value	primer f	primer r
Abaecin: LOC100631078	0.233	0.830	GCCACAATATGTGGAATCCT	ATGACCAGGGTTTGGTAATG
Alaserpin: LOC100648602	0.667	0.530	TGCTGAAATGCTAGATGACACG	GCATATCGCTCGTTAACTCAGG
Apidaecin: LOC100649867	0.025	0.981	CGTGTTGTGTGCAGTCGGTAATTC	GTCTGCGTGAAAGTTCAAGGCTTC
Argonaut 2: LOC100647036	0.930	0.397	AATTGCAAGATCAACCTGCC	CCTACCCAAAGACAAGGCAA
Aubergine: LOC100646008	0.145	0.890	GTCGCCCTTCTGCATATCTC	AAGATCGAACTGCTATCCGC
Basket:LOC100631091	1.239	0.285	GGAACAAGATAATCGAGCAACTG	CTGGCTTTCAATCGGTTGTG
BGRP1: LOC100647662	1.108	0.333	AACGTGGAAGTCAAAGATGG	GCGAACGATGACTTGGTATT
Decay: LOC100646875	2.611	0.063	AAGAAGACCTCGGTCCTTAGA	CAGCTGCAAATGAAGTAATGCG
Ferritin: LOC105666120	1.456	0.233	AAAGAATTGGACGCAAATGG	CAGCGAACTGATGTCCAAGA
Hymenoptaecin: LOC100631061	0.046	0.966	TTCATCGTACTGGCTCTCTTCTG	AGCCGTAGTATTCTTCCACAGC
PGRP-S1: LOC100651417	2.017	0.090	TTTCCATGTTGCTCGCTTCG	CGCGGTTTCCCTTTCGATATTAG
PGRP.L: LOC100648322	2.072	0.128	CAGCCACCTACGACAGATTT	GTACATTCCGCTTGTGTCCT
PGRP.S3: LOC100651268	0.774	0.477	CGTGAAGGAGCTCATACCAT	CCAGGACTCATAGTGGCTGT
PGRP.SC2: LOC105666394	1.729	0.149	TTGGTTGGCGAAGATGGAAAC	CGCGCTTGGATTATGACCAAC
Relish: LOC100631072	1.164	0.323	CAGCAGTAAAAATCCCCGAC	CAGCACGAATAAGTGAACATA
serpin27a: LOC100644115	1.086	0.354	CCGATCATCCATTCGTATTC	ACCTGCACTTGATATCCCTG
SPN_3.4A: LOC100648482	-2.312	0.066	GCAGAGACAAATGTTGAAGCAC	CACAGTCTGGGATAATGAAGAACC
SPN_3.4B: LOC100648717	-0.552	0.609	ATGGTGCTTTGTTCATCAGTCG	GACCCAATGACAGCAGTAACAG
tepA: LOC100631073	1.667	0.165	GCGTTCTATGACCACCTGTT	TACAGGTTACTCCACAGCCC
Transferrin: LOC100650460	0.277	0.794	CAATTTCTTCACCGCATCCT	CCTCGTTATTTGGCTTGCAT
Vitellogenin: LOC100649042	0.930	0.393	GTGACAAGCGAAGAGACTATTATG	CCGTGTTATCTGGCGTGAC
