# Supplementary material for: Royal Decree: Gene Expression in Trans-Generationally Immune Primed Bumblebee Workers Mimics a Primary Immune Response
Source: PLoS One. 2016 Jul 21;11(7):e0159635. doi: 10.1371/journal.pone.0159635 (PMC4956190; doi:10.1371/journal.pone.0159635)
Supplement: S2 Table — (PDF) [file pone.0159635.s008.pdf]

xloc	gene	sample_1	sample_2	status	FPKM_1	FPKM_2	log2_fold_ch	test_stat	p_value	q_value	significant
XLOC_000088	LOC100649912	NA_	NN	OK	36.1649	77.5923	1.10132	3.18328	5.00E-05	0.00345316	yes
XLOC_000103	LOC100642949	NA_	NN	OK	28.7527	17.1451	-0.745901	-2.06283	3.00E-04	0.015594	yes
XLOC_000112	LOC100645359	NA_	NN	OK	63.2226	37.9806	-0.735177	-2.08301	0.00025	0.0135134	yes
XLOC_000138	LOC100643639	NA_	NN	OK	41.5701	18.9828	-1.13086	-2.2712	4.00E-04	0.0197472	yes
XLOC_000139	LOC100644116	NA_	NN	OK	30.8348	4.16192	-2.88924	-3.75106	5.00E-05	0.00345316	yes
XLOC_000148	LOC100645819	NA_	NN	OK	99.0927	261.619	1.40062	3.86495	5.00E-05	0.00345316	yes
XLOC_000160	LOC100647603	NA_	NN	OK	37.4934	13.4829	-1.4755	-2.81572	5.00E-05	0.00345316	yes
XLOC_000233	LOC100645975,LOC100646094	NA_	NN	OK	25.0159	42.4212	0.761938	1.88154	9.00E-04	0.0374257	yes
XLOC_000245	LOC100648892	NA_	NN	OK	202.886	93.0689	-1.1243	-2.94237	5.00E-05	0.00345316	yes
XLOC_000297	LOC100648083	NA_	NN	OK	45.1809	82.6045	0.870509	2.47907	5.00E-05	0.00345316	yes
XLOC_000341	NA	NA_	NN	OK	45.8117	77.0146	0.749417	2.07243	0.00035	0.0178855	yes
XLOC_000450	LOC100643875	NA_	NN	OK	90.0471	30.8937	-1.54337	-2.56807	5.00E-05	0.00345316	yes
XLOC_000468	LOC100643149	NA_	NN	OK	587.442	1920.66	1.70908	3.07884	5.00E-05	0.00345316	yes
XLOC_000469	LOC100643108	NA_	NN	OK	1742.34	526.519	-1.72647	-2.63558	2.00E-04	0.0111481	yes
XLOC_000482	LOC100642553	NA_	NN	OK	76.9878	46.4857	-0.727842	-2.12067	0.00045	0.0216446	yes
XLOC_000496	LOC100644873	NA_	NN	OK	10.9412	6.19469	-0.820662	-2.16902	0.00015	0.00897192	yes
XLOC_000536	LOC100646612	NA_	NN	OK	27.9357	14.7883	-0.917656	-2.42585	1.00E-04	0.00631785	yes
XLOC_000562	NA	NA_	NN	OK	32.4062	15.8885	-1.02828	-1.90116	0.0012	0.0473458	yes
XLOC_000563	LOC100650142	NA_	NN	OK	15.1551	6.34424	-1.25629	-2.74552	5.00E-05	0.00345316	yes
XLOC_000596	LOC100647835	NA_	NN	OK	262.41	153.065	-0.777681	-2.14948	5.00E-05	0.00345316	yes
XLOC_000619	LOC100645702	NA_	NN	OK	84.0826	18.5336	-2.18167	-5.3669	5.00E-05	0.00345316	yes
XLOC_000643	LOC100643069	NA_	NN	OK	5.17589	2.77817	-0.897673	-1.83018	0.00125	0.0489743	yes
XLOC_000650	LOC100651433	NA_	NN	OK	34.2842	54.6663	0.673108	1.89494	0.001	0.0408069	yes
XLOC_000671	LOC100646372	NA_	NN	OK	23.7831	11.9313	-0.995192	-2.46244	5.00E-05	0.00345316	yes
XLOC_000686	LOC100650632	NA_	NN	OK	56.2456	21.4993	-1.38745	-2.87861	5.00E-05	0.00345316	yes
XLOC_000699	LOC100643233	NA_	NN	OK	13.239	3.85755	-1.77904	-2.42487	0.00025	0.0135134	yes
XLOC_000709	LOC100645433	NA_	NN	OK	8.53215	24.0767	1.49666	3.58653	5.00E-05	0.00345316	yes
XLOC_000712	LOC100646253	NA_	NN	OK	76.879	44.9033	-0.775767	-2.21091	0.00055	0.0255477	yes
XLOC_000713	LOC100646373	NA_	NN	OK	1113.86	1980.74	0.830476	1.99368	7.00E-04	0.0307351	yes
XLOC_000730	LOC100649684,LOC100649799	NA_	NN	OK	256.529	531.975	1.05223	2.63626	5.00E-05	0.00345316	yes
XLOC_000761	LOC100649801	NA_	NN	OK	137.819	232.417	0.753935	2.20269	3.00E-04	0.015594	yes
XLOC_000762	LOC100650111	NA_	NN	OK	287.2	1032.83	1.84648	4.68741	5.00E-05	0.00345316	yes
XLOC_000776	LOC100650592	NA_	NN	OK	34.2266	81.4044	1.24999	3.03857	5.00E-05	0.00345316	yes
XLOC_000795	LOC100645937	NA_	NN	OK	10.1536	3.82553	-1.40826	-2.01882	8.00E-04	0.0340602	yes
XLOC_000868	LOC100649615	NA_	NN	OK	156.128	99.7871	-0.645802	-1.78618	0.0017	0.0626688	no
XLOC_001055	LOC100647719	NA_	NN	OK	46.0732	124.819	1.43784	3.94388	5.00E-05	0.00345316	yes
XLOC_001057	LOC100648085	NA_	NN	OK	7.36291	4.299	-0.776276	-1.86881	0.0014	0.0532583	no
XLOC_001088	LOC100646374	NA_	NN	OK	33.0154	18.706	-0.819639	-2.3583	1.00E-04	0.00631785	yes
XLOC_001104	LOC100649988	NA_	NN	OK	60.0469	258.384	2.10536	2.38228	0.00025	0.0135134	yes
XLOC_001180	LOC100646893	NA_	NN	OK	42.4922	78.9404	0.893566	2.5258	5.00E-05	0.00345316	yes
XLOC_001241	LOC100648391	NA_	NN	OK	597.105	1250.65	1.06663	2.41618	5.00E-05	0.00345316	yes
XLOC_001309	LOC100642196	NA_	NN	OK	12.6924	8.01852	-0.66256	-1.93106	0.00065	0.0289604	yes
XLOC_001320	LOC100649872	NA_	NN	OK	64.5123	134.326	1.0581	1.89178	0.00195	0.0699171	no
XLOC_001366	NA	NA_	NN	OK	11.1345	60.9059	2.45155	4.42876	5.00E-05	0.00345316	yes
XLOC_001367	LOC100646942	NA_	NN	OK	8.79891	18.9872	1.10963	2.47716	1.00E-04	0.00631785	yes
XLOC_001394	LOC100643073	NA_	NN	OK	18.1929	10.3567	-0.812813	-2.20044	3.00E-04	0.015594	yes
XLOC_001402	PEPCK	NA_	NN	OK	7.28517	80.089	3.45857	7.7229	5.00E-05	0.00345316	yes
XLOC_001409	LOC100650031	NA_	NN	OK	8.78054	15.821	0.849459	1.82875	0.0015	0.0563533	no
XLOC_001410	LOC100649919	NA_	NN	OK	7.47961	14.71	0.975758	2.12398	3.00E-04	0.015594	yes
XLOC_001424	LOC100644009	NA_	NN	OK	37.5087	16.9133	-1.14906	-2.28855	2.00E-04	0.0111481	yes
XLOC_001475	LOC100631078	NA_	NN	OK	17758.9	176.515	-6.6526	-10.2647	5.00E-05	0.00345316	yes
XLOC_001524	LOC100642680	NA_	NN	OK	107.509	35.6547	-1.59229	-4.1568	5.00E-05	0.00345316	yes
XLOC_001601	LOC100644839	NA_	NN	OK	139.678	241.839	0.79194	1.97868	0.0014	0.0532583	no
XLOC_001602	LOC100644713	NA_	NN	OK	253.998	140.852	-0.850637	-2.19511	2.00E-04	0.0111481	yes
XLOC_001631	NA	NA_	NN	OK	23.9543	14.5802	-0.716271	-2.10322	7.00E-04	0.0307351	yes
XLOC_001638	LOC100646895	NA_	NN	OK	49.4352	83.31	0.752951	1.99756	6.00E-04	0.0273847	yes
XLOC_001695	LOC100645624	NA_	NN	OK	79.765	47.4501	-0.749345	-1.96032	0.00045	0.0216446	yes
XLOC_001720	LOC100644799	NA_	NN	OK	7.60584	13.4294	0.820216	2.12769	0.00045	0.0216446	yes
XLOC_001738	LOC100650271	NA_	NN	OK	24.8304	44.9207	0.855271	2.37473	0.00015	0.00897192	yes
XLOC_001740	LOC100649764	NA_	NN	OK	39.9057	70.2852	0.816627	2.17184	3.00E-04	0.015594	yes
XLOC_001747	LOC100648625	NA_	NN	OK	37.4869	4.0379	-3.21471	-5.2535	5.00E-05	0.00345316	yes
XLOC_001792	LOC100649617	NA_	NN	OK	8.89311	24.5723	1.46627	3.68087	5.00E-05	0.00345316	yes
XLOC_001796	LOC100650678	NA_	NN	OK	13.9119	6.48602	-1.10091	-2.73652	5.00E-05	0.00345316	yes
XLOC_001834	LOC100645983	NA_	NN	OK	15.5262	9.96753	-0.639399	-1.73946	0.00205	0.072445	no
XLOC_001962	LOC100651716	NA_	NN	OK	105.389	20.1782	-2.38485	-5.28665	5.00E-05	0.00345316	yes
XLOC_001977	LOC100646701	NA_	NN	OK	161.725	285.409	0.81949	2.21543	1.00E-04	0.00631785	yes
XLOC_002035	LOC100648933	NA_	NN	OK	4.15763	9.85023	1.24439	2.29826	2.00E-04	0.0111481	yes
XLOC_002038	LOC100642443	NA_	NN	OK	13798.6	192.525	-6.16334	-10.1642	5.00E-05	0.00345316	yes
XLOC_002054	LOC100642321	NA_	NN	OK	38.6292	63.7733	0.723259	1.86805	0.0019	0.0685623	no
XLOC_002073	LOC100649725	NA_	NN	OK	5.27713	2.1109	-1.3219	-2.67351	5.00E-05	0.00345316	yes
XLOC_002076	LOC100642442	NA_	NN	OK	110.711	30.1485	-1.87664	-5.22725	5.00E-05	0.00345316	yes
XLOC_002090	LOC100646102	NA_	NN	OK	13.03	5.56462	-1.22749	-2.15217	4.00E-04	0.0197472	yes
XLOC_002097	LOC100647494	NA_	NN	OK	62.4577	38.0447	-0.715184	-2.04811	7.00E-04	0.0307351	yes
XLOC_002106	LOC100645439	NA_	NN	OK	3.9184	1.47368	-1.41084	-2.06617	5.00E-05	0.00345316	yes
XLOC_002125	LOC100645190	NA_	NN	OK	3.16763	11.215	1.82396	3.02342	5.00E-05	0.00345316	yes

XLOC_002155	LOC100649207	NA_	NN	OK	57.6856	19.4528	-1.56823	-4.40884	5.00E-05	0.00345316	yes
XLOC_002177	VSP	NA_	NN	OK	714.886	178.686	-2.00029	-5.45478	5.00E-05	0.00345316	yes
XLOC_002178	LOC100642484	NA_	NN	OK	7.17387	72.5504	3.33816	5.06608	5.00E-05	0.00345316	yes
XLOC_002180	LOC100652274	NA_	NN	OK	14.3656	70.8352	2.30185	5.28543	5.00E-05	0.00345316	yes
XLOC_002181	LOC100652157	NA_	NN	OK	21.6833	77.6753	1.84087	4.35993	5.00E-05	0.00345316	yes
XLOC_002182	LOC100652036	NA_	NN	OK	6.59034	36.2327	2.45887	4.4957	5.00E-05	0.00345316	yes
XLOC_002183	LOC100651916	NA_	NN	OK	42.1512	232.48	2.46346	6.29643	5.00E-05	0.00345316	yes
XLOC_002187	LOC100650999	NA_	NN	OK	10.0223	5.13668	-0.964305	-2.06995	0.00035	0.0178855	yes
XLOC_002189	NA	NA_	NN	OK	44.0079	16.1225	-1.44869	-2.85326	5.00E-05	0.00345316	yes
XLOC_002191	NA	NA_	NN	OK	688.435	3232.34	2.23119	2.84739	5.00E-05	0.00345316	yes
XLOC_002192	LOC100650318	NA_	NN	OK	75.386	48.0179	-0.650726	-1.82708	0.0016	0.0596539	no
XLOC_002219	LOC100643115	NA_	NN	OK	2109.01	708.973	-1.57276	-3.5926	5.00E-05	0.00345316	yes
XLOC_002226	LOC100652273	NA_	NN	OK	7.16726	2.63609	-1.44302	-2.31337	4.00E-04	0.0197472	yes
XLOC_002267	LOC100642684	NA_	NN	OK	5.96195	0.364454	-4.03198	-3.61601	0.00035	0.0178855	yes
XLOC_002269	LOC100642921	NA_	NN	OK	222.717	104.564	-1.09083	-3.08922	5.00E-05	0.00345316	yes
XLOC_002299	LOC100651675	NA_	NN	OK	12.0638	6.73673	-0.840561	-2.10429	2.00E-04	0.0111481	yes
XLOC_002394	LOC100649375	NA_	NN	OK	64.3413	41.2587	-0.641049	-1.81798	0.00175	0.0640314	no
XLOC_002417	LOC100643929	NA_	NN	OK	14.4415	5.00473	-1.52886	-2.82987	5.00E-05	0.00345316	yes
XLOC_002585	LOC100649054	NA_	NN	OK	38.3653	20.122	-0.931025	-2.10333	4.00E-04	0.0197472	yes
XLOC_002599	LOC100642803	NA_	NN	OK	132.234	213.715	0.692592	2.02051	5.00E-04	0.0235311	yes
XLOC_002649	LOC100644638,LOC100644886	NA_	NN	OK	1210.44	305.54	-1.9861	-3.61882	5.00E-05	0.00345316	yes
XLOC_002650	NA	NA_	NN	OK	177.808	19.2463	-3.20767	-5.73953	5.00E-05	0.00345316	yes
XLOC_002659	LOC100642635,LOC100642754	NA_	NN	OK	60.4266	35.6291	-0.762129	-2.07513	0.00045	0.0216446	yes
XLOC_002665	LOC100651196	NA_	NN	OK	33.2348	74.0817	1.15642	2.91283	5.00E-05	0.00345316	yes
XLOC_002714	LOC100647727	NA_	NN	OK	22.1191	11.0408	-1.00245	-2.19081	4.00E-04	0.0197472	yes
XLOC_002729	LOC100643887	NA_	NN	OK	5.67709	3.1043	-0.870881	-1.79904	0.00205	0.072445	no
XLOC_002765	LOC100645002	NA_	NN	OK	122.277	228.7	0.903299	2.56895	5.00E-05	0.00345316	yes
XLOC_002767	LOC100644169	NA_	NN	OK	292.74	479.361	0.711493	2.00067	5.00E-04	0.0235311	yes
XLOC_002780	LOC100644884	NA_	NN	OK	2.50169	7.34231	1.55333	2.45791	1.00E-04	0.00631785	yes
XLOC_002787	LOC100647216	NA_	NN	OK	418.102	1178.11	1.49455	3.94217	5.00E-05	0.00345316	yes
XLOC_002825	LOC100644718	NA_	NN	OK	20.1546	12.0802	-0.738459	-2.00942	7.00E-04	0.0307351	yes
XLOC_002874	LOC100648673	NA_	NN	OK	90.6501	174.373	0.943795	2.55935	5.00E-05	0.00345316	yes
XLOC_002876	LOC100645987	NA_	NN	OK	107.737	31.7342	-1.7634	-4.57594	5.00E-05	0.00345316	yes
XLOC_002880	LOC100648940	NA_	NN	OK	73.6804	43.9686	-0.744808	-2.18766	3.00E-04	0.015594	yes
XLOC_002989	LOC100648437	NA_	NN	OK	23.9929	6.75564	-1.82844	-2.97841	5.00E-05	0.00345316	yes
XLOC_002997	LOC100645752	NA_	NN	OK	19.9481	8.6986	-1.1974	-2.28446	2.00E-04	0.0111481	yes
XLOC_003014	LOC100651521	NA_	NN	OK	228.255	356.848	0.644665	1.86171	0.0013	0.0504803	no
XLOC_003020	LOC100643077	NA_	NN	OK	18.7976	9.40286	-0.999378	-2.23796	1.00E-04	0.00631785	yes
XLOC_003028	Def	NA_	NN	OK	23740.8	63.7044	-8.54176	-11.516	5.00E-05	0.00345316	yes
XLOC_003038	LOC100644846	NA_	NN	OK	9.04079	0.25965	-5.12181	-5.94539	5.00E-05	0.00345316	yes
XLOC_003064	LOC100643324	NA_	NN	OK	8.33859	20.037	1.26479	3.17432	5.00E-05	0.00345316	yes
XLOC_003097	LOC100648747	NA_	NN	OK	6.69938	3.83036	-0.806548	-1.79779	0.0015	0.0563532	no
XLOC_003143	LOC100643448,LOC100643573	NA_	NN	OK	109.373	197.287	0.851043	2.22072	5.00E-05	0.00345316	yes
XLOC_003147	LOC100651996	NA_	NN	OK	272.976	442.76	0.697752	1.95232	8.00E-04	0.0340602	yes
XLOC_003312	LOC100649997	NA_	NN	OK	74.2975	119.82	0.689486	2.02018	4.00E-04	0.0197472	yes
XLOC_003350	LOC100651567	NA_	NN	OK	3.77557	0.644897	-2.54955	-3.3099	5.00E-05	0.00345316	yes
XLOC_003351	LOC100651683	NA_	NN	OK	107.073	11.9385	-3.1649	-7.75307	5.00E-05	0.00345316	yes
XLOC_003356	LOC100642454	NA_	NN	OK	182.727	285.028	0.641408	1.81206	0.0017	0.0626688	no
XLOC_003409	LOC100652120	NA_	NN	OK	9.37045	3.39471	-1.46483	-2.09479	0.00075	0.0324233	yes
XLOC_003430	LOC100642250	NA_	NN	OK	184.36	113.921	-0.694493	-1.9234	0.0011	0.0442897	yes
XLOC_003489	LOC100645755	NA_	NN	OK	6.51484	16.2332	1.31714	2.878	5.00E-05	0.00345316	yes
XLOC_003557	LOC100649576	NA_	NN	OK	37.5184	23.8179	-0.655553	-1.90116	6.00E-04	0.0273847	yes
XLOC_003608	LOC100642844	NA_	NN	OK	24.7664	9.69116	-1.35364	-3.65278	5.00E-05	0.00345316	yes
XLOC_003637	LOC100645125	NA_	NN	OK	2.80575	9.32954	1.73342	3.19073	5.00E-05	0.00345316	yes
XLOC_003643	LOC100642209	NA_	NN	OK	12.1132	1.06391	-3.50912	-4.60742	5.00E-05	0.00345316	yes
XLOC_003665	LOC100647066	NA_	NN	OK	71.9281	119.212	0.728907	1.96677	0.00075	0.0324233	yes
XLOC_003677	LOC100650884	NA_	NN	OK	23.8988	12.9489	-0.884101	-2.46115	5.00E-05	0.00345316	yes
XLOC_003697	LOC100644966	NA_	NN	OK	2561.66	907.195	-1.4976	-3.46023	5.00E-05	0.00345316	yes
XLOC_003698	LOC100645083	NA_	NN	OK	5.88069	1.80109	-1.70711	-2.71667	5.00E-05	0.00345316	yes
XLOC_003735	LOC100644134	NA_	NN	OK	75.2079	122.927	0.708848	2.05848	0.00045	0.0216446	yes
XLOC_003738	LOC100649731	NA_	NN	OK	52.9427	18.4256	-1.52272	-4.31828	5.00E-05	0.00345316	yes
XLOC_003823	LOC100644562,LOC100645524	NA_	NN	OK	308.066	840.34	1.44773	3.00711	5.00E-05	0.00345316	yes
XLOC_003827	LOC100646382	NA_	NN	OK	65.7415	35.1939	-0.901481	-2.07226	0.00045	0.0216446	yes
XLOC_003829	LOC100646624	NA_	NN	OK	13.9544	33.2857	1.25418	3.20519	5.00E-05	0.00345316	yes
XLOC_003830	LOC100646863	NA_	NN	OK	4.11131	0.776268	-2.40497	-2.58676	0.00015	0.00897192	yes
XLOC_003860	LOC100645991	NA_	NN	OK	4.23789	2.12044	-0.998981	-1.8625	0.00165	0.0611699	no
XLOC_003889	LOC100648399	NA_	NN	OK	3.00624	0.624608	-2.26694	-3.53233	5.00E-05	0.00345316	yes
XLOC_003919	LOC100646625	NA_	NN	OK	58.5795	38.142	-0.619017	-1.78666	0.002	0.0713194	no
XLOC_003922	LOC100648166	NA_	NN	OK	9.69794	19.3115	0.993706	2.28713	5.00E-05	0.00345316	yes
XLOC_003933	LOC100650726	NA_	NN	OK	26.8008	9.02985	-1.5695	-3.51106	5.00E-05	0.00345316	yes
XLOC_003952	LOC100651882	NA_	NN	OK	163.331	82.4559	-0.9861	-2.84064	5.00E-05	0.00345316	yes
XLOC_003964	LOC100648209	NA_	NN	OK	2.94569	0.839328	-1.8113	-2.48852	0.00015	0.00897192	yes
XLOC_004023	LOC100644369	NA_	NN	OK	26.6745	5.88545	-2.18024	-4.04282	5.00E-05	0.00345316	yes
XLOC_004024	LOC100649580	NA_	NN	OK	43.9096	4.18523	-3.39116	-5.98455	5.00E-05	0.00345316	yes
XLOC_004025	LOC100644683	NA_	NN	OK	2063.28	256.663	-3.007	-4.89164	5.00E-05	0.00345316	yes
XLOC_004075	NA	NA_	NN	OK	3.274	7.0158	1.09955	1.84875	0.00195	0.0699171	no

XLOC_004076	NA	NA_	NN	OK	4.56515	11.5365	1.33748	2.26939	5.00E-04	0.0235311	yes
XLOC_004108	LOC100646028	NA_	NN	OK	318.724	175.323	-0.862297	-1.99465	6.00E-04	0.0273847	yes
XLOC_004165	NA	NA_	NN	OK	10.2795	119.747	3.54215	6.76716	5.00E-05	0.00345316	yes
XLOC_004205	LOC100642761	NA_	NN	OK	223.07	90.5062	-1.30141	-3.57144	5.00E-05	0.00345316	yes
XLOC_004206	LOC100648752	NA_	NN	OK	773.969	403.421	-0.939988	-2.54214	5.00E-05	0.00345316	yes
XLOC_004227	LOC100647974	NA_	NN	OK	118.814	19.4938	-2.60762	-7.03073	5.00E-05	0.00345316	yes
XLOC_004358	LOC100644893	NA_	NN	OK	49.5373	93.6654	0.919002	2.61488	5.00E-05	0.00345316	yes
XLOC_004445	LOC100648790	NA_	NN	OK	15.6828	31.2001	0.99237	2.52258	5.00E-05	0.00345316	yes
XLOC_004512	LOC100642574,LOC100647662	NA_	NN	OK	466.416	252.419	-0.8858	-1.88684	0.0017	0.0626688	no
XLOC_004671	LOC100646385	NA_	NN	OK	29.8346	61.5301	1.0443	2.87997	5.00E-05	0.00345316	yes
XLOC_004702	LOC100648828	NA_	NN	OK	39.0381	61.5665	0.657265	1.9338	0.001	0.0408069	yes
XLOC_004716	LOC100645995	NA_	NN	OK	24.537	14.6427	-0.744779	-2.01057	3.00E-04	0.015594	yes
XLOC_004724	LOC100646348	NA_	NN	OK	723.46	326.535	-1.14768	-2.47447	5.00E-05	0.00345316	yes
XLOC_004736	LOC100648521	NA_	NN	OK	79.4721	39.1898	-1.01997	-2.84038	5.00E-05	0.00345316	yes
XLOC_004784	LOC100651647,LOC100652317	NA_	NN	OK	39.0516	78.1488	1.00084	2.25186	5.00E-05	0.00345316	yes
XLOC_004811	LOC100645839,LOC100645951	NA_	NN	OK	14.9432	7.6341	-0.968958	-1.88281	0.0015	0.0563532	no
XLOC_004812	LOC100646065	NA_	NN	OK	4.22371	0	#NAME?	NA	5.00E-05	0.00345316	yes
XLOC_004835	LOC100643535	NA_	NN	OK	17.6348	9.86948	-0.837382	-2.09324	3.00E-04	0.015594	yes
XLOC_004865	LOC100649218	NA_	NN	OK	4.10083	1.18824	-1.78709	-2.54377	2.00E-04	0.0111481	yes
XLOC_004950	LOC100645916	NA_	NN	OK	17.2594	7.80883	-1.1442	-2.19001	0.00025	0.0135134	yes
XLOC_004999	LOC100651889	NA_	NN	OK	42.3475	17.151	-1.30398	-3.45594	5.00E-05	0.00345316	yes
XLOC_005039	LOC100652247	NA_	NN	OK	33.1281	14.9358	-1.14928	-3.01327	5.00E-05	0.00345316	yes
XLOC_005062	LOC100646668	NA_	NN	OK	4.42196	1.96841	-1.16766	-1.8878	0.002	0.0713194	no
XLOC_005075	LOC100643580	NA_	NN	OK	85.2796	50.1589	-0.765695	-2.22903	5.00E-05	0.00345316	yes
XLOC_005118	LOC100644530	NA_	NN	OK	15.3126	9.79609	-0.644443	-1.826	0.00165	0.0611699	no
XLOC_005161	LOC100651766	NA_	NN	OK	8.68915	3.60793	-1.26804	-2.40904	2.00E-04	0.0111481	yes
XLOC_005162	LOC100649338	NA_	NN	OK	1018.23	383.345	-1.40935	-2.69441	5.00E-05	0.00345316	yes
XLOC_005240	LOC100644450	NA_	NN	OK	409.097	181.324	-1.17388	-3.1139	5.00E-05	0.00345316	yes
XLOC_005282	LOC100643900	NA_	NN	OK	33.1376	13.9597	-1.2472	-2.46732	5.00E-05	0.00345316	yes
XLOC_005325	LOC100643169	NA_	NN	OK	25.824	14.924	-0.791074	-2.15488	5.00E-05	0.00345316	yes
XLOC_005395	LOC100643086	NA_	NN	OK	80.5671	159.194	0.982523	2.87582	5.00E-05	0.00345316	yes
XLOC_005406	LOC100650166	NA_	NN	OK	25.5178	47.1484	0.885703	2.37711	5.00E-05	0.00345316	yes
XLOC_005408	LOC100650692	NA_	NN	OK	79.5176	47.2954	-0.749576	-2.08865	0.00025	0.0135134	yes
XLOC_005426	LOC100645456	NA_	NN	OK	29.7817	15.4662	-0.945303	-2.56959	5.00E-05	0.00345316	yes
XLOC_005512	LOC100645956	NA_	NN	OK	207.209	340.898	0.718255	1.96393	0.00065	0.0289604	yes
XLOC_005517	LOC100649221,LOC100649341	NA_	NN	OK	90.7289	56.8247	-0.675043	-1.88379	0.00135	0.0517066	no
XLOC_005553	NA	NA_	NN	OK	552.618	314.798	-0.811855	-2.13672	4.00E-04	0.0197472	yes
XLOC_005555	LOC100648717	NA_	NN	OK	579.006	280.993	-1.04304	-2.83613	5.00E-05	0.00345316	yes
XLOC_005610	LOC100644732	NA_	NN	OK	20.5159	57.4111	1.48459	3.63852	5.00E-05	0.00345316	yes
XLOC_005663	LOC100649304	NA_	NN	OK	8.97173	27.057	1.59254	4.0817	5.00E-05	0.00345316	yes
XLOC_005683	LOC100642892	NA_	NN	OK	40.1345	62.1084	0.629946	1.83723	0.00135	0.0517066	no
XLOC_005691	LOC100647906	NA_	NN	OK	20.0605	10.2507	-0.968642	-2.67048	5.00E-05	0.00345316	yes
XLOC_005736	LOC100642500	NA_	NN	OK	33.759	17.1916	-0.973571	-2.19635	3.00E-04	0.015594	yes
XLOC_005738	LOC100649306	NA_	NN	OK	15.3683	6.4378	-1.25532	-2.3323	1.00E-04	0.00631785	yes
XLOC_005745	LOC100649106	NA_	NN	OK	47.554	21.0719	-1.17425	-3.15023	5.00E-05	0.00345316	yes
XLOC_005750	LOC100647229	NA_	NN	OK	8.52791	17.1704	1.00966	2.2141	0.00025	0.0135134	yes
XLOC_005821	LOC100647110	NA_	NN	OK	7.78759	3.30177	-1.23794	-2.46979	1.00E-04	0.00631785	yes
XLOC_005873	LOC100648645	NA_	NN	OK	85.9255	35.1515	-1.2895	-3.64312	5.00E-05	0.00345316	yes
XLOC_005968	LOC100651170	NA_	NN	OK	209.424	329.678	0.654633	1.83897	0.00175	0.0640314	no
XLOC_006112	LOC100651094	NA_	NN	OK	6289.04	1134.05	-2.47135	-3.43798	5.00E-05	0.00345316	yes
XLOC_006148	LOC100651578	NA_	NN	OK	22.2772	6.97005	-1.67632	-2.62963	5.00E-05	0.00345316	yes
XLOC_006206	LOC100645340	NA_	NN	OK	4.85039	0.293673	-4.04582	-3.75135	7.00E-04	0.0307351	yes
XLOC_006244	LOC100645920	NA_	NN	OK	20.1323	9.05247	-1.15313	-2.09222	0.0012	0.0473458	yes
XLOC_006258	LOC100645022,LOC100645214	NA_	NN	OK	172.729	287.107	0.733076	2.04548	0.00055	0.0255477	yes
XLOC_006264	LOC100643779	NA_	NN	OK	92.5196	174.237	0.913218	2.32846	5.00E-05	0.00345316	yes
XLOC_006297	LOC100646239	NA_	NN	OK	54.5066	32.6907	-0.737549	-2.01996	0.00035	0.0178855	yes
XLOC_006305	LOC100644101	NA_	NN	OK	0.897822	9.41978	3.39119	3.26921	3.00E-04	0.015594	yes
XLOC_006314	LOC100642295	NA_	NN	OK	36.2327	10.1645	-1.83376	-4.49087	5.00E-05	0.00345316	yes
XLOC_006344	LOC100644692	NA_	NN	OK	69.7119	43.5481	-0.678793	-1.94689	0.00085	0.0357628	yes
XLOC_006345	NA	NA_	NN	OK	69.0754	141.86	1.03823	2.62606	5.00E-05	0.00345316	yes
XLOC_006437	LOC100645806	NA_	NN	OK	18.8276	6.94093	-1.43965	-3.02896	5.00E-05	0.00345316	yes
XLOC_006470	LOC100643254	NA_	NN	OK	52.9337	92.8813	0.811203	2.01571	0.00045	0.0216446	yes
XLOC_006471	LOC100643622	NA_	NN	OK	20.2966	48.4103	1.25407	3.4436	5.00E-05	0.00345316	yes
XLOC_006482	LOC100645885	NA_	NN	OK	96.1614	233.065	1.2772	3.73725	5.00E-05	0.00345316	yes
XLOC_006490	LOC100646873	NA_	NN	OK	92.7041	175.673	0.922186	2.57483	5.00E-05	0.00345316	yes
XLOC_006509	LOC100650336,LOC100650454	NA_	NN	OK	84.6302	145.295	0.77974	2.09041	2.00E-04	0.0111481	yes
XLOC_006579	LOC100645650	NA_	NN	OK	62.4048	146.608	1.23224	3.57753	5.00E-05	0.00345316	yes
XLOC_006664	LOC100648294	NA_	NN	OK	75.5935	36.1049	-1.06607	-2.9793	5.00E-05	0.00345316	yes
XLOC_006677	LOC100647588	NA_	NN	OK	45.7398	91.9328	1.00713	2.81842	5.00E-05	0.00345316	yes
XLOC_006685	LOC100644817	NA_	NN	OK	64.632	147.383	1.18925	2.74842	5.00E-05	0.00345316	yes
XLOC_006698	LOC100644616	NA_	NN	OK	20.9739	11.2349	-0.900615	-2.38626	5.00E-05	0.00345316	yes
XLOC_006735	LOC100644939	NA_	NN	OK	28.9924	14.054	-1.04469	-2.54909	5.00E-05	0.00345316	yes
XLOC_006757	LOC100645023	NA_	NN	OK	2.36033	0.670804	-1.81502	-2.34976	2.00E-04	0.0111481	yes
XLOC_006761	LOC100644375	NA_	NN	OK	53.1939	85.3061	0.681387	1.84087	0.0015	0.0563532	no
XLOC_006765	LOC100643258	NA_	NN	OK	1.97124	0.505691	-1.96277	-2.70051	5.00E-05	0.00345316	yes
XLOC_006839	LOC100648029	NA_	NN	OK	102.058	203.89	0.998401	2.82957	5.00E-05	0.00345316	yes

XLOC_006881	LOC100651655	NA_	NN	OK	310.817	97.3056	-1.67547	-4.54904	5.00E-05	0.00345316	yes
XLOC_006886	LOC100643588	NA_	NN	OK	288.148	90.8557	-1.66516	-4.56027	5.00E-05	0.00345316	yes
XLOC_006894	LOC100645536	NA_	NN	OK	6.11819	3.15335	-0.956218	-1.83278	0.00175	0.0640314	no
XLOC_006914	NA	NA_	NN	OK	24.7942	6.96101	-1.83263	-4.54472	5.00E-05	0.00345316	yes
XLOC_006924	LOC100648224	NA_	NN	OK	126.276	24.4024	-2.37148	-6.18365	5.00E-05	0.00345316	yes
XLOC_006949	LOC100652292	NA_	NN	OK	38.4147	74.4546	0.954703	2.47495	5.00E-05	0.00345316	yes
XLOC_006963	LOC100651851	NA_	NN	OK	122.71	424.172	1.78939	2.27731	0.00065	0.0289604	yes
XLOC_006964	LOC100651730	NA_	NN	OK	11.9659	67.8554	2.50353	3.82712	5.00E-05	0.00345316	yes
XLOC_006986	LOC100642897	NA_	NN	OK	2.69057	1.17333	-1.1973	-1.82943	0.00195	0.0699171	no
XLOC_007010	LOC100644779	NA_	NN	OK	17.6348	9.59438	-0.878159	-2.33551	5.00E-05	0.00345316	yes
XLOC_007055	LOC100651261	NA_	NN	OK	44.5178	27.9482	-0.671625	-1.88759	0.00135	0.0517066	no
XLOC_007058	LOC100651731	NA_	NN	OK	164.883	97.471	-0.758398	-2.04616	0.00045	0.0216446	yes
XLOC_007076	LOC100647116	NA_	NN	OK	55.3698	32.7235	-0.758775	-2.02282	0.00075	0.0324233	yes
XLOC_007126	LOC100648607	NA_	NN	OK	21.099	2.13765	-3.30308	-5.81395	5.00E-05	0.00345316	yes
XLOC_007127	LOC100648799	NA_	NN	OK	31.7085	17.8261	-0.830876	-2.08385	4.00E-04	0.0197472	yes
XLOC_007128	LOC100649711	NA_	NN	OK	16.9713	2.54582	-2.7369	-3.7825	5.00E-05	0.00345316	yes
XLOC_007136	LOC100647787	NA_	NN	OK	12.0804	6.93801	-0.800075	-1.89524	0.00105	0.0425819	yes
XLOC_007236	LOC100650219	NA_	NN	OK	104.541	172.964	0.726409	1.86462	0.00135	0.0517066	no
XLOC_007238	LOC100642940	NA_	NN	OK	6.72282	16.7976	1.32112	1.88548	0.00155	0.0580099	no
XLOC_007296	LOC100646517	NA_	NN	OK	23.0162	41.7751	0.859995	1.92078	9.00E-04	0.0374257	yes
XLOC_007329	LOC100648144,LOC100648258	NA_	NN	OK	68.9356	31.4319	-1.13302	-2.37054	0.00085	0.0357628	yes
XLOC_007354	LOC100646359	NA_	NN	OK	16.8405	33.4242	0.988956	2.80634	5.00E-05	0.00345316	yes
XLOC_007386	LOC100643057	NA_	NN	OK	3.67193	1.21476	-1.59587	-1.85797	0.0017	0.0626688	no
XLOC_007389	LOC100644497	NA_	NN	OK	2.02707	0	#NAME?	NA	5.00E-05	0.00345316	yes
XLOC_007393	LOC100644978	NA_	NN	OK	22.2605	1.28916	-4.10998	-5.47261	5.00E-05	0.00345316	yes
XLOC_007500	LOC100642226	NA_	NN	OK	286.5	33.5367	-3.09472	-8.49535	5.00E-05	0.00345316	yes
XLOC_007508	LOC100650012	NA_	NN	OK	16.7433	30.9817	0.887835	2.17451	3.00E-04	0.015594	yes
XLOC_007640	LOC100650221	NA_	NN	OK	143.399	63.493	-1.17537	-3.26147	5.00E-05	0.00345316	yes
XLOC_007644	LOC100650789	NA_	NN	OK	302.193	191.415	-0.658766	-1.93032	0.00075	0.0324233	yes
XLOC_007647	LOC100646603	NA_	NN	OK	190.036	313.343	0.72147	2.00827	0.00045	0.0216446	yes
XLOC_007673	LOC100643060	NA_	NN	OK	11.9023	7.01983	-0.76173	-1.91489	0.00115	0.0457868	yes
XLOC_007701	LOC100645349	NA_	NN	OK	56.0173	260.389	2.21673	6.01595	5.00E-05	0.00345316	yes
XLOC_007727	LOC100650132	NA_	NN	OK	20.4252	38.3453	0.908695	2.61633	5.00E-05	0.00345316	yes
XLOC_007755	LOC100643345	NA_	NN	OK	15.627	8.8084	-0.827085	-2.12154	4.00E-04	0.0197472	yes
XLOC_007770	LOC100649429	NA_	NN	OK	8.09588	14.9235	0.882327	2.21529	5.00E-05	0.00345316	yes
XLOC_007807	NA	NA_	NN	OK	4.45356	1.46901	-1.60012	-2.07405	0.001	0.0408069	yes
XLOC_007848	LOC100647555	NA_	NN	OK	24.0044	64.8729	1.43432	2.78926	5.00E-05	0.00345316	yes
XLOC_007862	LOC100643830	NA_	NN	OK	12.6479	5.03933	-1.32759	-3.41526	5.00E-05	0.00345316	yes
XLOC_007877	LOC100648148	NA_	NN	OK	5.27523	22.361	2.08368	2.75407	1.00E-04	0.00631785	yes
XLOC_007919	LOC100645892	NA_	NN	OK	288.863	442.659	0.615809	1.74453	0.00205	0.072445	no
XLOC_007943	LOC100650460	NA_	NN	OK	5214.24	759.907	-2.77856	-3.477	5.00E-05	0.00345316	yes
XLOC_008038	LOC100644380	NA_	NN	OK	88.5163	41.4661	-1.09401	-3.147	5.00E-05	0.00345316	yes
XLOC_008056	LOC100651143	NA_	NN	OK	188.826	296.179	0.649411	1.90799	7.00E-04	0.0307351	yes
XLOC_008082	LOC100644824	NA_	NN	OK	15.0824	9.01961	-0.741729	-1.9867	8.00E-04	0.0340602	yes
XLOC_008131	LOC100645424	NA_	NN	OK	60.8983	7.39256	-3.04226	-4.64655	5.00E-05	0.00345316	yes
XLOC_008163	LOC100647082	NA_	NN	OK	530.949	135.265	-1.97278	-5.53218	5.00E-05	0.00345316	yes
XLOC_008190	LOC100642903	NA_	NN	OK	326.083	576.411	0.821857	2.25885	5.00E-05	0.00345316	yes
XLOC_008240	LOC100644946	NA_	NN	OK	22.7078	13.2575	-0.776374	-2.17767	0.00015	0.00897192	yes
XLOC_008241	LOC100644582	NA_	NN	OK	4.80511	0.457982	-3.39121	-4.01668	5.00E-05	0.00345316	yes
XLOC_008242	LOC100648536	NA_	NN	OK	613.032	1.25599	-8.93099	-9.71852	5.00E-05	0.00345316	yes
XLOC_008264	LOC100648036	NA_	NN	OK	3.28911	14.098	2.09972	3.75063	5.00E-05	0.00345316	yes
XLOC_008294	LOC100644984	NA_	NN	OK	3.32915	0.963444	-1.78888	-2.11234	0.00135	0.0517066	no
XLOC_008307	LOC100649276	NA_	NN	OK	40.4575	64.5549	0.67412	1.87654	0.0013	0.0504803	no
XLOC_008310	LOC100649601	NA_	NN	OK	13.0199	33.8551	1.37865	3.40365	5.00E-05	0.00345316	yes
XLOC_008312	LOC100650863	NA_	NN	OK	1.22997	3.2629	1.40754	1.94908	0.0018	0.0656165	no
XLOC_008314	LOC100650745	NA_	NN	OK	2.97545	177.167	5.89586	10.4448	5.00E-05	0.00345316	yes
XLOC_008375	LOC100651465	NA_	NN	OK	30.7961	51.331	0.737085	2.0867	2.00E-04	0.0111481	yes
XLOC_008454	LOC100649787	NA_	NN	OK	46.9792	109.661	1.22295	3.2269	5.00E-05	0.00345316	yes
XLOC_008636	LOC100644828	NA_	NN	OK	39.1379	76.285	0.962833	2.72693	5.00E-05	0.00345316	yes
XLOC_008640	LOC100650704	NA_	NN	OK	69.4458	173.71	1.32272	3.61849	5.00E-05	0.00345316	yes
XLOC_008664	LOC100643262	NA_	NN	OK	95.259	15.499	-2.61968	-3.77124	5.00E-05	0.00345316	yes
XLOC_008676	LOC100648346	NA_	NN	OK	69.1943	142.223	1.03943	2.67111	5.00E-05	0.00345316	yes
XLOC_008695	LOC100650705	NA_	NN	OK	25.6443	13.5075	-0.924875	-1.79513	0.00205	0.072445	no
XLOC_008761	LOC100643788	NA_	NN	OK	114.29	37.7342	-1.59875	-4.33421	5.00E-05	0.00345316	yes
XLOC_008780	LOC100649157	NA_	NN	OK	23.1598	47.4762	1.03558	2.79831	5.00E-05	0.00345316	yes
XLOC_008828	LOC100642546	NA_	NN	OK	4.70581	1.2598	-1.90125	-2.13621	5.00E-04	0.0235311	yes
XLOC_008897	NA	NA_	NN	OK	7.86494	3.40003	-1.20989	-2.09411	5.00E-04	0.0235311	yes
XLOC_008909	LOC100643142	NA_	NN	OK	15.4275	32.3032	1.06617	2.45372	5.00E-05	0.00345316	yes
XLOC_008954	LOC100649239	NA_	NN	OK	15.1636	5.91487	-1.35819	-2.29484	2.00E-04	0.0111481	yes
XLOC_008988	LOC100646640	NA_	NN	OK	11.757	4.679	-1.32925	-2.02221	0.00075	0.0324233	yes
XLOC_009013	LOC100644867	NA_	NN	OK	140.704	291.392	1.0503	2.86319	5.00E-05	0.00345316	yes
XLOC_009060	LOC100643869	NA_	NN	OK	89.4505	162.104	0.857756	2.38303	5.00E-05	0.00345316	yes
XLOC_009065	LOC100644539	NA_	NN	OK	5.87723	0.586406	-3.32516	-2.86531	0.00085	0.0357628	yes
XLOC_009130	LOC100652301	NA_	NN	OK	1879.69	931.451	-1.01294	-2.36338	5.00E-05	0.00345316	yes
XLOC_009190	LOC100647796	NA_	NN	OK	953.05	2367.89	1.31298	2.61208	5.00E-05	0.00345316	yes
XLOC_009245	LOC100646642	NA_	NN	OK	23.8783	54.9457	1.2023	2.57012	5.00E-05	0.00345316	yes

XLOC_009253	LOC100648002	NA_	NN	OK	36.428	125.233	1.7815	4.72219	5.00E-05	0.00345316	yes
XLOC_009304	LOC100642713	NA_	NN	OK	65.6354	118.129	0.847816	2.2692	1.00E-04	0.00631785	yes
XLOC_009340	LOC100649907	NA_	NN	OK	393.443	888.616	1.17541	3.08057	5.00E-05	0.00345316	yes
XLOC_009350	LOC100651509	NA_	NN	OK	58.5838	30.7163	-0.931497	-2.27667	5.00E-05	0.00345316	yes
XLOC_009357	LOC100652332	NA_	NN	OK	3.51427	0.792022	-2.14961	-2.14484	0.00185	0.0671899	no
XLOC_009385	LOC100651905	NA_	NN	OK	14.7823	30.6706	1.05299	2.79148	5.00E-05	0.00345316	yes
XLOC_009465	LOC100645228	NA_	NN	OK	12.3154	7.13858	-0.78675	-2.23119	5.00E-05	0.00345316	yes
XLOC_009466	LOC100645662	NA_	NN	OK	433.882	237.172	-0.871368	-2.32686	5.00E-05	0.00345316	yes
XLOC_009468	LOC100645782	NA_	NN	OK	74.3685	37.1771	-1.00028	-2.78882	5.00E-05	0.00345316	yes
XLOC_009485	NA	NA_	NN	OK	24.3548	8.70058	-1.48502	-2.01239	0.00125	0.0489743	yes
XLOC_009495	LOC100647883	NA_	NN	OK	63.5773	112.403	0.822096	2.24626	5.00E-05	0.00345316	yes
XLOC_009505	LOC100642272	NA_	NN	OK	781.236	1439.51	0.881746	2.07719	0.00055	0.0255477	yes
XLOC_009566	LOC100649867	NA_	NN	OK	12341	390.313	-4.98268	-9.87853	5.00E-05	0.00345316	yes
XLOC_009568	LOC100649983	NA_	NN	OK	26.8607	10.5002	-1.35508	-3.04472	5.00E-05	0.00345316	yes
XLOC_009579	LOC100651307	NA_	NN	OK	361.785	866.083	1.25937	3.32495	5.00E-05	0.00345316	yes
XLOC_009604	LOC100644470	NA_	NN	OK	65.1029	457.027	2.81148	5.10547	5.00E-05	0.00345316	yes
XLOC_009617	LOC100642789	NA_	NN	OK	52.0531	26.2362	-0.988427	-2.14062	2.00E-04	0.0111481	yes
XLOC_009834	NA	NA_	NN	OK	109.717	182.255	0.732168	2.12216	0.00025	0.0135134	yes
XLOC_009843	LOC100645510	NA_	NN	OK	8.217	0.883309	-3.21762	-3.44769	1.00E-04	0.00631785	yes
XLOC_009860	LOC100646646	NA_	NN	OK	54.8109	28.9349	-0.921652	-2.38796	5.00E-05	0.00345316	yes
XLOC_009862	LOC100648041	NA_	NN	OK	18.6115	9.85122	-0.91782	-2.08597	0.00025	0.0135134	yes
XLOC_009885	LOC100650989	NA_	NN	OK	7.8314	2.686	-1.54381	-2.4883	1.00E-04	0.00631785	yes
XLOC_009940	NA	NA_	NN	OK	5958.82	206.382	-4.85164	-10.9525	5.00E-05	0.00345316	yes
XLOC_009958	NA	NA_	NN	OK	73014.9	37.7272	-10.9184	-15.3104	5.00E-05	0.00345316	yes
