# Supplementary material for: Royal Decree: Gene Expression in Trans-Generationally Immune Primed Bumblebee Workers Mimics a Primary Immune Response
Source: PLoS One. 2016 Jul 21;11(7):e0159635. doi: 10.1371/journal.pone.0159635 (PMC4956190; doi:10.1371/journal.pone.0159635)
Supplement: S3 Table — (PDF) [file pone.0159635.s009.pdf]

xloc	gene	sample_1	sample_2	status	FPKM_1	FPKM_2	log2_fold_change	test_stat	p_value	q_value	significant
XLOC_000619	LOC100645702	AN	NN	OK	36.6008	18.5336	-0.981733	-2.47206	5.00E-05	0.00345316	yes
XLOC_001088	LOC100646374	AN	NN	OK	38.6835	18.706	-1.04822	-2.95426	5.00E-05	0.00345316	yes
XLOC_001402	PEPCK	AN	NN	OK	29.5708	80.089	1.43743	3.74494	5.00E-05	0.00345316	yes
XLOC_001475	LOC100631078	AN	NN	OK	2383.16	176.515	-3.75501	-5.38066	5.00E-05	0.00345316	yes
XLOC_001602	LOC100644713	AN	NN	OK	332.765	140.852	-1.24032	-3.40058	5.00E-05	0.00345316	yes
XLOC_002038	LOC100642443	AN	NN	OK	1761.24	192.525	-3.19348	-4.58661	5.00E-05	0.00345316	yes
XLOC_002178	LOC100642484	AN	NN	OK	20.8605	72.5504	1.79821	3.63521	5.00E-05	0.00345316	yes
XLOC_002183	LOC100651916	AN	NN	OK	594.206	232.48	-1.35385	-2.79615	5.00E-05	0.00345316	yes
XLOC_002219	LOC100643115	AN	NN	OK	390.75	708.973	0.859484	2.21551	1.00E-04	0.00631785	yes
XLOC_003028	Def	AN	NN	OK	2724.13	63.7044	-5.41826	-7.22478	5.00E-05	0.00345316	yes
XLOC_003351	LOC100651683	AN	NN	OK	24.6678	11.9385	-1.04701	-2.55888	5.00E-05	0.00345316	yes
XLOC_003637	LOC100645125	AN	NN	OK	4.01029	9.32954	1.2181	2.34286	1.00E-04	0.00631785	yes
XLOC_004999	LOC100651889	AN	NN	OK	36.9364	17.151	-1.10675	-2.85579	5.00E-05	0.00345316	yes
XLOC_005282	LOC100643900	AN	NN	OK	31.4235	13.9597	-1.17057	-2.72443	5.00E-05	0.00345316	yes
XLOC_006478	LOC100644816	AN	NN	OK	75.5648	20.4398	-1.88633	-2.92659	5.00E-05	0.00345316	yes
XLOC_006924	LOC100648224	AN	NN	OK	87.2479	24.4024	-1.8381	-4.46132	5.00E-05	0.00345316	yes
XLOC_008131	LOC100645424	AN	NN	OK	27.9538	7.39256	-1.9189	-3.07779	5.00E-05	0.00345316	yes
XLOC_008828	LOC100642546	AN	NN	OK	5.6473	1.2598	-2.16437	-2.63025	5.00E-05	0.00345316	yes
XLOC_009566	LOC100649867	AN	NN	OK	1284.4	390.313	-1.71839	-3.5509	5.00E-05	0.00345316	yes
XLOC_009940	NA	AN	NN	OK	702.397	206.382	-1.76697	-3.80947	5.00E-05	0.00345316	yes
XLOC_009958	NA	AN	NN	OK	4719.13	37.7272	-6.96677	-9.31693	5.00E-05	0.00345316	yes
