# Supplementary material for: Royal Decree: Gene Expression in Trans-Generationally Immune Primed Bumblebee Workers Mimics a Primary Immune Response
Source: PLoS One. 2016 Jul 21;11(7):e0159635. doi: 10.1371/journal.pone.0159635 (PMC4956190; doi:10.1371/journal.pone.0159635)
Supplement: S4 Table — (PDF) [file pone.0159635.s010.pdf]

Gene ID	NCBI Accession	Name	F primer	R primer	Note
LOC100645702	XM_003393468	Kynurenine/alpha-aminoadipate aminotransferase	ATCCTAGCAAACCATGCAACGC	CCATTGCCAAGTTCATTTCTTCGC	
LOC100646374	XM_003393788	Battenin	TTCTTGGTGGTGGAGCATACGTG	TACGATCCGCCCTTGGAATCTC	
PEPCK	XM_003394476	Phosphoenolpyruvate carboxykinase	ATGGCGCTACCAACCCTAATGC	TGCCTCGCCAATCGGTGATTTC	
LOC100644713	XM_003394095	Laccase-2	TCCAGGGTGGTGGTTACTTCATTG	CGTGTATGACCAGTTCCATTCCAG	
LOC100642484	XM_003395038	Venom Protease	ACGTTTCTCAGGAGTGATGAGGAC	TCAACTGATGGATGCAGGAGGAC	
LOC100643115	XM_003394962	Hypothetical protein LOC100643115	GAAGCTGCAAGCGTACGGAAAC	AACCGCTTCGCTGCCACTTATG	
LOC100651889	XM_003398271	Hypothetical protein LOC100651889	ATACTTGTTGGCCGAGCGTTGC	GAGTTGCGTCTTCCCTTGTTCTTG	
LOC100643900	XM_003398287	15-hydroxyprostaglandin dehydrogenase [NAD+]-like	GTGTGCCCTGGACGTACAAATAC	TTTCGCCACACACTCTGGTG	
LOC100644816	XM_003399422	Mast cell degranulating peptide (MCDP)	ACCCGATTTGTATAAGAGTCTGT	TCCTAGAGCTTGTTGGAATTTAGT	
LOC100648224	XM_003400005	Uncharacterized LOC100648224	GGAGGAATTCGCTGAGCAGTTG	TCGGTTGCTCTCTGTGTTCTTAGG	
LOC100648499	XR_132448	Apidaecin	CGTGTTGTGTGCAGTCGGTAATTC	GTCTGCGTGAAAGTTCAAGGCTTC	
LOC100642442	XM_003394713	BGRP2	TAACTCCCTTTGGAAACACG	GGCGGTAAAATACTGAACGA	
LOC100647662	XM_003397996	BGRP1	AACGTGGAAGTCAAAGATGG	GCGAACGATGACTTGGTATT	
LOC100649867	XM_003402966	Apidaecin	CCCGACTAATGTACCTGCCA	GAAGGTGCGAATGTGTTGGA	
Def	XM_003395924	Defensin	GTCTGCCTTTGTCGCAAGAC	GACATTAGTCGCGTCTTCTTCG	LOC100631063
LOC100631061	XR_132450	Hymenoptaecin	TTCATCGTACTGGCTCTCTTCTG	AGCCGTAGTATTCTTCCACAGC	
LOC100631078	XM_003394653	Abaecin	GCCACAATATGTGGAATCCT	ATGACCAGGGTTTGGTAATG	
LOC100631072	XM_003399472	Relish	CAGCAGTAAAAATCCCCGAC	CAGCACGAATAAGTGAACATA	
dl	XM_003402459	Dorsal	GCTGATGAAGTGAAAGAGGC	GCTTGGTCCTGCGTTTATTA	
LOC100631062	HM142999	PPO	AGCGGCATAATACGTTGTGT	CCGAGGGATAGAAAGTCTCC	
LOC100651268	XM_003401893	PGRP-SA	CGTGAAGGAGCTCATACCAT	CCAGGACTCATAGTGGCTGT	PGRP 2 like (NCBI name)
LOC100747500	XM_003493213	PGRP-SC2	TTGGTTGGCGAAGATGGAAAC	CGCGCTTGGATTATGACCAAC	Based on Bombus impatiens sequence
LOC100651417	XM_003400112	PGRP-S1	TTTCCATGTTGCTCGCTTCG	CGCGGTTTCCCTTTCGATATTAG	PGRP-LB (NCBI name)
LOC100650460	XM_003401163	Transferrin	CAATTTCTTCACCGCATCCT	CCTCGTTATTTGGCTTGCAT	
LOC100648482	XM_003399138	SPN3/4A	GCAGAGACAAATGTTGAAGCAC	CACAGTCTGGGATAATGAAGAACC	
LOC100631084	XM_003401944	Ef-1a-f2	GCTGGTGACTCGAAGAACAATC	GGGTGGTTCAACACAATAACCTG	Housekeeping
LOC100631077	XM_003400700	RPL13	GGTTTAACCAGCCAGCTAGAAA	CTTCACAGGTCTTGGTGCAA	Housekeeping
