# Supplementary material for: Royal Decree: Gene Expression in Trans-Generationally Immune Primed Bumblebee Workers Mimics a Primary Immune Response
Source: PLoS One. 2016 Jul 21;11(7):e0159635. doi: 10.1371/journal.pone.0159635 (PMC4956190; doi:10.1371/journal.pone.0159635)
Supplement: S5 Table — Cells highlighted in yellow are significant at p < 0.05, those highlighted in blue approach significance p < 0.1. (PDF) [file pone.0159635.s011.pdf]

Target	Induced analysis (NA vs NN)				Memory analysis (AN vs NN)			
	Y-J_lambda	t_6	p	Fold_expression	Y-J_lambda	t_6	p	Fold_expression
Hymenoptaecin: LOC100631061	0.585	-47.384	0.000000	300.014	1.673	2.908	0.027040	4.352
Defensin: LOC100631063	0.488	-25.265	0.000000	131.152	1.272	2.821	0.030315	5.906
Abaecin: LOC100631078	0.522	-11.688	0.000024	18.749	1.39	3.177	0.019138	2.860
Apidaecin: LOC100649867	0.637	-14.474	0.000007	18.382	1.514	1.828	0.117387	2.804
Transferrin: LOC100650460	0.547	-10.615	0.000041	10.679	0.656	2.509	0.045959	1.864
BGRP2: LOC100642442	-0.839	-5.234	0.001949	5.000	-0.765	3.349	0.015435	4.618
BGRP1: LOC100647662	0.431	-3.988	0.007219	2.299	1.064	1.932	0.101497	1.302
SPN3/4A: LOC100648482	0.177	-4.171	0.005869	2.074	0.439	0.633	0.549958	1.370
Apidaecin: LOC100648499	4.407	-3.820	0.008765	1.646	8.528	-0.070	0.946285	1.018
Relish: LOC100631072	-1.033	-5.002	0.002449	1.629	1.631	0.649	0.540523	1.089
Aminotransferase: LOC100645702	1.03	-0.744	0.485144	1.489	1.78	0.865	0.420175	1.457
PGRP-S1: LOC100651417	1.54	-2.371	0.055475	1.436	0.987	1.415	0.206920	1.232
LOC100648224	1.03	-0.658	0.535038	1.381	0.479	2.115	0.078856	1.880
Dorsal: LOC100631054	1.354	-0.562	0.594669	1.330	-1.209	1.899	0.106334	1.435
LOC100651889	-2.101	-2.169	0.073118	1.316	-0.429	1.447	0.198015	1.196
PGRP-SC2: LOC100747500(Bimp)	0.335	-2.096	0.080885	1.288	0.933	1.611	0.158290	1.309
LOC100643115	0.877	-0.521	0.620898	1.260	0.837	0.306	0.770234	1.139
Laccase-2: LOC100644713	1.235	-1.008	0.352488	1.152	1.195	2.710	0.035108	1.533
PPO: LOC100631062	-1.583	-0.336	0.748651	1.114	-2.641	0.520	0.621849	1.021
Battenin: LOC100646374	4.602	-0.105	0.919467	1.060	1.855	1.726	0.135185	1.332
PGRP-SA: LOC100651268	0.346	-0.134	0.897876	1.057	0.014	0.444	0.672339	1.117
NAD+: LOC100643900	2.066	0.615	0.560909	0.823	0.711	0.524	0.619233	1.147
LOC100644816	0.619	0.770	0.470253	0.529	0.047	0.759	0.476532	1.697
Venom protease:LOC100642484	1.082	1.793	0.123222	0.337	0.567	-1.389	0.214171	0.289
PEPCK: LOC100631088	2.16	2.654	0.037843	0.263	1.252	-1.416	0.206626	0.471
